# Sick of eating: Eco-evo-immuno dynamics of predators and their trophically acquired parasites

**DOI:** 10.1111/evo.14353

**Published:** 2021-10-12

**Authors:** Samuel R. Fleischer, Daniel I. Bolnick, Sebastian J. Schreiber

**Affiliations:** 1 Graduate Group in Applied Mathematics, University of California, Davis, Davis California 95616; 3 Department of Ecology and Evolutionary Biology, University of Connecticut, Storrs Connecticut 06269; 4 Department of Evolution and Ecology, University of California, Davis, Davis California 95616

**Keywords:** Apparent competition, ecoevolutionary feedbacks, parasitism, predation, quantitative genetics, trade-offs

## Abstract

When predators consume prey, they risk becoming infected with their prey’s parasites, which can then establish the predator as a secondary host. A predator population’s diet therefore influences what parasites it is exposed to, as has been repeatedly shown in many species such as threespine stickleback (*Gasterosteus aculeatus*) (more benthic-feeding individuals obtain nematodes from oligocheate prey, whereas limnetic-feeding individuals catch cestodes from copepod prey). These differing parasite encounters, in turn, determine how natural selection acts on the predator’s immune system. We might therefore expect that ecoevolutionary dynamics of a predator’s diet (as determined by its ecomorphology) should drive correlated evolution of its immune traits. Conversely, the predator’s immunity to certain parasites might alter the relative costs and benefits of different prey, driving evolution of its ecomorphology. To evaluate the potential for ecological morphology to drive evolution of immunity, and vice versa, we use a quantitative genetics framework coupled with an ecological model of a predator and two prey species (the diet options). Our analysis reveals fundamental asymmetries in the evolution of ecomorphology and immunity. When ecomorphology rapidly evolves, it determines how immunity evolves, but not vice versa. Weak trade-offs in ecological morphology select for diet generalists despite strong immunological trade-offs, but not vice versa. Only weak immunological trade-offs can explain negative diet-infection correlations across populations. The analysis also reveals that eco-evo-immuno feedbacks destabilize population dynamics when trade-offs are sufficiently weak and heritability is sufficiently high. Collectively, these results highlight the delicate interplay between multivariate trait evolution and the dynamics of ecological communities.

Community ecologists are increasingly interested in the impact of parasites on communities (Anderson and Sukhdeo 2011; Sukhdeo 2012; Wood and Johnson 2015), altering both food web dynamics and structure (Lafferty et al. 2006; Cortez and Weitz 2014; van Velzen and Gaedke 2017). These community and ecosystem effects of parasites will depend on the type and intensity of infections. Hosts’ infection status is regulated by two sequential processes: “encounter filters” regulate the frequency with which various parasites gain access to a prospective host, and subsequently “compatibility filters” determine whether the infection succeeds. As an example of an encounter filter, many parasites have complex life cycles and are transmitted upward through a food chain. Predators acquire such parasites by consuming infected prey (Iritani and Sato 2018; Rogawa et al. 2018), so a host’s position within the food web will dictate what kinds of parasites it is exposed to. Prey availability and predator foraging rate affect the frequency of those exposure events. Only after a parasite gains access to a host do compatibility filters (e.g., the host’s immune system or the parasite’s ability to access host resources) become relevant to whether the parasite survives to infect the host or is eliminated.

Disease ecologists often treat encounter and compatibility filters as independent processes, the former in the domain of community ecology, the latter a concern of molecular biology and immunology. The goal of this article is to challenge this distinction using theory to illustrate some ways in which ecoevolutionary dynamics will lead to correlated evolution of otherwise independent traits affecting host-parasite encounter and compatibility. Although foraging ecology and immunity may reflect genetically distinct phenotypes, they may evolve in response to the same underlying selective pressures. In particular, we investigate the potential for a reciprocal eco-evo-immuno feedback. The evolution of dietary traits can change the fitness landscape for immune traits by altering encounter rates with prey-acquired parasites. Conversely, the evolution of immunity can change the fitness landscape for dietary traits by altering the cost-benefit balance for alternative prey.

Consider the interaction between threespine stickleback (*Gasterosteus aculeatus*), a small fish found in north temperate coastal habitats: estuaries, rivers, and lakes. In lakes, stickleback have access to a mix of large benthic invertebrates or smaller mid-water (limnetic) zooplankton. The relative availabilities of benthic versus limnetic prey vary with lake size: larger lakes contain relatively more open water habitat compared to shallow benthic substrate. Therefore, stickleback diet covaries with lake size, with corresponding morphological adaptations (Lavin and McPhail 1985, 1986); populations in small lakes tend to eat more benthic prey and have evolved fewer, shorter gill rakers, larger body depth, and wider gape, whereas populations in larger lakes tend to eat more limnetic prey, and have evolved more, longer fill rakers, greater suction feeding capacity, and more fusiform body shape. In intermediate-sized lakes, stickleback are morphologically and ecologically intermediate, and exhibit appreciable among-individual diet variation along the same benthic-limnetic axis seen between lakes (Snowberg et al. 2015; Bolnick and Ballare 2020). Because of the complex trophically transmitted lifecycle of many parasites, this ecologically driven variation in diet affects sticklebacks’ parasite communities (Bolnick et al. 2020a, b). The cestode *Schistocephalus solidus* uses cyclopoid copepods (a limnetic prey) as a first host. In contrast, the nematode *Eustronglyides* sp. uses benthic-dwelling oligocheate worms as their primary host. As a result, stickleback morphology and diet are correlated with parasite intake rates: individuals consuming more limnetic copepods have higher (*S. solidus*) infection rates, whereas more benthic individuals have more nematodes (Stutz et al. 2014).

We therefore expect that sticklebacks’ diet should dictate how selection acts on immune traits, as different parasite exposure rates should favor different pathogen-recognition or effector responses. This immune evolution might negate or even reverse the observable correlation between population mean diet and infection prevalence. Stutz et al. (2014) found that benthic-transmitted nematode prevalence was lowest in the benthic populations, and highest in limnetic populations—the reverse of the trend we expect a priori and observe among individuals within populations. Stutz et al. (2014) suggested that correlated evolution of diet and immunity might be the cause of this negative correlation.

Associations between ecomorphology, diet, and infection risk are not unique to lake stickleback. California sea otters exhibit persistent among-individual differences in foraging preferences for alternative prey; individuals that prefer marine snails have high prevalence of Toxoplasma parasites, whereas abalone-foragers have generally lower infection rates (Estes et al. 2003; Tinker et al. 2008; Johnson et al. 2009). In Lake Tanganyikan cichlids (Hayward et al. 2017), and Lake Tana barbs (Sibbing et al. 1998), populations specializing on different kinds of prey exhibit consistently different parasite infections. Thus, given the empirically widespread phenomenon of trophically transmitted parasites, we should broadly expect that dietary preferences drive infection rates and immune evolution in many species.

Conversely, infection rates can drive evolution of diet. When infection risk is tied to certain prey, the net benefit of that prey depends on parasite prevalence and host immunity. Hosts can evolve avoidance behaviors to reduce parasite encounter rates (Behringer et al. 2018; Weinstein et al. 2018), leading to diet shifts that then favor new morphological adaptations. Hablützel et al. (2017) argue that this avoidance process may have driven the evolution of diatom-specialization in Tropheini cichlids, which harbor fewer parasites than their more generalist relatives. In an opinion article, Britton and Andreou (2016) argue that parasites may often favor the evolution of host diet specialization. But avoidance is not the only option: the evolution of greater immunity by a predator can reduce the harmful effects of parasites, potentially enabling a diet shift to add a formerly hazardous prey. Ecomorphology may then subsequently evolve to optimize attack efficiency on this newly safe diet.

We therefore expect that foraging ecology can drive immune evolution, and vice versa, resulting in a reciprocal feedback between ecology and immunity. Such eco-evo-immuno feedbacks may explain the reversal of diet-infection correlations at different spatial scales (e.g., more benthic individuals carry more nematodes, but more benthic populations have lower nematode prevalence, Stutz et al. 2014). Motivated by this potential feedback loop, we seek to address three questions. First, under what conditions is there a reciprocal feedback between diet and immune evolution? If these conditions do not hold, when does the evolution of the predator’s diet determine the evolution of its immune system, or vice versa? Second, how are ecomorphology and immune trait values correlated spatiotemporally? Is there a relationship between these traits’ selection pressures even if they are genetically uncorrelated? Finally, when does the joint evolution of diet and immune traits, along with predator-prey dynamics, obscure the relationship between parasite exposure risk and actual infection rates? As hypothesized by Stutz et al. (2014), evolution of predator immune traits may negate or even reverse an expected positive correlation between intake of, and infection by, a particular parasite. Thus, populations frequently exposed to particular parasites may have lower infection rates than populations that are rarely exposed (and hence susceptible) to the few parasites they do encounter. To answer these questions, we analyze a model of a community of two prey species sharing a predator with two evolving quantitative traits determining diet and defense against prey-specific parasites.

## Models

Let *P* = *P*(*t*) be the density of a predator population and *N*_*i*_ = *N*_*i*_(*t*) be the densities of prey populations *i* for *i* = 1,2. Each prey species experiences logistic growth in the absence of the predator, with intrinsic growth rates *r*_*i*_ and carrying capacities *K*_*i*_. The predator species has a per-capita death rate *d*, attacks prey species *i* with attack rate *a*_*i*_, and converts food into offspring with efficiency *b*_*i*_.

A percentage *c*_*i*_ of prey *i* individuals are infected with parasite *i* that decreases predator fecundity by *m*_*i*_*S*_*i*_, where *m*_*i*_ is the maximal negative effect of parasite *i* and *S*_*i*_ ≤ 1 is a measure of predator susceptibility to parasite *i*. Low *S*_*i*_ corresponds to a strong immunity to parasite *i*. Then the ecological dynamics are given by

$$\begin{array}{c} \frac{\text{d}P}{\text{d}t}=P[\left(b_{1}-c_{1}m_{1}S_{1}\right)a_{1}N_{1}+\left(b_{2}-c_{2}m_{2}S_{2}\right)a_{2}N_{2}-d],\;\frac{\text{d}N_{i}}{\text{d}t}=N_{i}\left[r_{i}\left(1-\frac{N_{i}}{K_{i}}\right)-a_{i}P\right],\;i=1,2. \end{array}$$

For the purpose of this article, we mostly treat parasite infection rates within the prey, *c*_*i*_, as a constant. This is biologically plausible when the parasite’s reproduction is not strongly limited by the infection rate in terminal hosts in the focal community, such as when most parasite propagules are imported from other patches in a metapopulation rather than locally produced. This is likely the case for parasites such as *Schistocephalus*, which are broadly dispersed by their avian terminal hosts. To evaluate how sensitive our results are to this assumption, we also formulated a model where the parasite load dynamically depends on predator consumption of prey and their susceptibility to infection.The formulation of this model, and a numerical exploration ofits dynamics, are presented in Supporting Information Appendix F. A discussion of the implications of these results are in the Discussion section.

The attack rate of the predator on prey *i* is determined by a quantitative trait *x*, for example, an ecomorphological trait. Attack rate of prey species *i* is maximal when *x* = θ_*i*_, where θ_*i*_ is the optimum trait to consume prey *i*, and decreases in a Gaussian manner as |*x* − θ_*i*_| increases (as in Schreiber et al. 2011). Specifically, the attack rate *a*_*i*_(*x*) on prey *i* equals

$$a_{i}(x)=\alpha_{i}\exp\left[-\frac{{\left(x-\theta_{i}\right)}^{2}}{2\zeta_{i}^{2}}\right],$$

where α_*i*_ is the maximal successful attack rate on prey *i*, and *ζ*_*i*_ determines how quickly attack rates decay with suboptimal ecomorphology of an individual. The smaller *ζ*_*i*_, the more phenotypically specialized a predator must be to use prey *i*. Thus, predator populations with greater *ζ*_*i*_ values experience less pressure to evolve morphological specialization.

The susceptibility of the predator to infection by parasite *i* is determined by a quantitative trait *y*. Susceptibility is minimized when *y* = φ_*i*_, where φ_*i*_ is the optimum trait to resist parasite *i*, and increases in a Gaussian manner as |*y* − φ_*i*_| increases. Specifically, the susceptibility *S*_*i*_(*y*) to parasite *i* equals

$$S_{i}(y)=\beta_{i}-\left(\beta_{i}-\gamma_{i}\right)\exp\left[-\frac{{\left(y-\phi_{i}\right)}^{2}}{2\tau_{i}^{2}}\right],$$

where β_*i*_ ≤ 1 and γ_*i*_ < β_*i*_ are the maximum and minimum susceptibility to parasite *i*, respectively, and τ_*i*_ determines how quickly rates of susceptibility to infection increase with suboptimal immunology of an individual. The smaller τ_*i*_, the more immunologically specialized a predator must be to significantly reduce susceptibility to infection by parasite *i*. Thus, predator populations with greater τ_*i*_ values experience less pressure to evolve immunological specialization. We have in mind a model of constitutively expressed innate immunity rather than adaptive immunity that is induced and grows following initial exposure.

The per-capita growth rate *W* of a predator with ecomorphology *x* and immunity *y* is given by

$$W\left(x,y,P,N_{1},N_{2}\right)=\left(b_{1}-c_{1}m_{1}S_{1}(y)\right)a_{1}(x)N_{1}\;\;+\left(b_{2}-c_{2}m_{2}S_{2}(y)\right)a_{2}(x)N_{2}-d$$

and the per-capita growth rate *Y*_*i*_ of prey interacting with predators with ecomorphology *x* is given by

$$Y_{i}\left(x,P,N_{1},N_{2}\right)=r_{i}\left(1-\frac{N_{i}}{K_{i}}\right)-a_{i}(x)P,\;i=1,2.$$

We assume the predator traits *x* and *y* are genetically independent and normally distributed over the population with means $\bar{x}$ and $\bar{y}$, respectively, and with $\sigma_{x}^{2}$ and $\sigma_{y}^{2}$, the total phenotypic variances of traits *x* and *y*, respectively. Let $\sigma_{x}^{2}=\sigma_{x,G}^{2}+\sigma_{x,E}^{2}$, where $\sigma_{x,G}^{2}$ is the phenotypic variation of trait *x* due to genotype and $\sigma_{x,E}^{2}$ is the phenotypic variation of trait *x* due to environmental conditions. Similarly, let $\sigma_{y}^{2}=\sigma_{y,G}^{2}+\sigma_{y,E}^{2}$. Here, we omit genetic-by-environmental interactions for mathematical simplicity, though these are common for both trophic and immunological traits. Note that the environmental variance component is not adaptive plasticity (e.g., not directionally dictated by prey availability or parasite exposure experience).

Integrating across the predator distribution of phenotypes, the average per-capita growth rate of the predator population $\bar{W}$ equals

$$\bar{W}\left(\bar{x},\bar{y},P,N_{1},N_{2}\right)=\left(b_{1}-c_{1}m_{1}{\bar{S}}_{1}(\bar{y})\right){\bar{a}}_{1}(\bar{x})N_{1}\;\;+\left(b_{2}-c_{2}m_{2}{\bar{S}}_{2}(\bar{y})\right){\bar{a}}_{2}(\bar{x})N_{2}-d,$$

where ${\bar{a}}_{i}$ and ${\bar{S}}_{i}$ are the averaged attack rate and susceptibility:

$${\bar{a}}_{i}(\bar{x})=\frac{\alpha_{i}\zeta_{i}}{\sqrt{\sigma_{x}^{2}+\zeta_{i}^{2}}}\exp\left[-\frac{{\left(\bar{x}-\theta_{i}\right)}^{2}}{2\left(\sigma_{x}^{2}+\zeta_{i}^{2}\right)}\right],\;i=1,2,\;{\bar{S}}_{i}(\bar{y})=\beta_{i}-\left(\beta_{i}-\gamma_{i}\right)\frac{\tau_{i}}{\sqrt{\sigma_{y}^{2}+\tau_{i}^{2}}}\exp\left[-\frac{{\left(\bar{y}-\phi_{i}\right)}^{2}}{2\left(\sigma_{y}^{2}+\tau_{i}^{2}\right)}\right],\;i=1,2.$$

The average per-capita growth rate ${\bar{Y}}_{i}$ of the prey *i* population is given by

$${\bar{Y}}_{i}\left(\bar{x},P,N_{1},N_{2}\right)=r_{i}\left(1-\frac{N_{i}}{K_{i}}\right)-{\bar{a}}_{i}(\bar{x})N_{i},\;i=1,2.$$

These functions describe the ecological dynamics:

(1a) $$\frac{\text{d}P}{\text{d}t}=P\bar{W}(\bar{x},\bar{y},P,N_{1},N_{2}),\;\frac{\text{d}N_{i}}{\text{d}t}=N_{i}{\bar{Y}}_{i}\left(\bar{x},P,N_{1},N_{2}\right),\;i=1,2.$$

See Supporting Information Appendix A for additional details regarding the model formulation.

Provided the predator trait distributions remain normal with constant variance over time, Lande (1976) showed that the change in mean trait over a single generation (in the absence of frequency-dependent selection) is proportional to the partial derivative of the predator population per-capita growth rate $\bar{W}$ with respect to that mean trait. The constants of proportionality are the portions of phenotypic variance due to genetic variation. We assume the morphological and immunological trains are genetically independent, and thus the evolutionary dynamics of $\bar{x}$ and $\bar{y}$ are given by

(1b) $$\frac{\text{d}\bar{x}}{\text{d}t}=\sigma_{x,G}^{2}\frac{\partial \bar{W}}{\partial \bar{x}}\;\frac{\text{d}\bar{y}}{\text{d}t}=\sigma_{y,G}^{2}\frac{\partial \bar{W}}{\partial \bar{y}},$$

where

$$\frac{\partial \bar{W}}{\partial \bar{x}}=\left(b_{1}-m_{1}c_{1}{\bar{S}}_{1}(\bar{y})\right){\bar{a}}_{1}(\bar{x})N_{1}\frac{\theta_{1}-\bar{x}}{\sigma_{x}^{2}+\zeta_{1}^{2}}\;\;+\left(b_{2}-m_{2}c_{2}{\bar{S}}_{2}(\bar{y})\right){\bar{a}}_{2}(\bar{x})N_{2}\frac{\theta_{2}-\bar{x}}{\sigma_{x}^{2}+\zeta_{2}^{2}},$$

$$\frac{\partial \bar{W}}{\partial \bar{y}}=m_{1}c_{1}{\bar{a}}_{1}(\bar{x})N_{1}\left(\beta_{1}-{\bar{S}}_{1}(\bar{y})\right)\frac{\phi_{1}-\bar{y}}{\sigma_{y}^{2}+\tau_{1}^{2}}\;\;+m_{2}c_{2}{\bar{a}}_{2}(\bar{x})N_{2}\left(\beta_{2}-{\bar{S}}_{2}(\bar{y})\right)\frac{\phi_{2}-\bar{y}}{\sigma_{y}^{2}+\tau_{2}^{2}}.$$

All model symbols are listed with a short description in Table 1.

## Methods

We use four numerical and analytical approaches to explore the questions posed in the introduction: (i) numerical simulations of population and evolutionary dynamics, (ii) analytical results obtained in the limit of slow evolution (low heritability) and timescale differences between the evolution of the two traits, (iii) Latin hypercube sampling across parameter space to analyze the effect of model parameters on simulation outcomes, and (iv) numerical approximations of Lyapnuov exponents to determine conditions for stable or chaotic dynamical behavior.

### NUMERICAL SIMULATIONS

We used standard numerical integration techniques (RungeKutta 4(5) with adaptive step size using Python’s scipy.integrate.odeint (Jones et al. 2001)) to simulate the models (1,1b) and (2). Parameters are given in Supporting Information Appendix B.

Lyapunov exponents describe how nearby trajectories behave in relation to a reference trajectory (Sprott 2003). Given an initial condition, a positive (negative) Lypunov exponent means that nearby trajectories on average move away from (toward) the reference trajectory, indicating chaos (stability) (Supporting Information Appendix C). We calculated Lyapunov exponents for full-model simulations over a two-dimensional subset of parameter space (σ_*y*,*G*_ vs. τ) to determine how the evolution of the immune trait *y* affects the ecoevolutionary dynamics. We also prove conditions for permanence of the system (all trajectories with positive initial condition eventually remain bounded away from the boundary *N*_1_*N*_2_*P*) in Supporting Information Appendix H.

### ANALYTIC REDUCTIONS FOR SLOW-EVOLUTION DYNAMICS

When the trait dynamics evolve at a sufficiently slower timescale than ecological dynamics, we can reduce the five-dimensional system to a two-dimensional system. This occurs, for example, if ecomorphological and immunity traits are only marginally heritable (small $\sigma_{x,G}^{2}/\sigma_{x}^{2}$ and $\sigma_{y,G}^{2}/\sigma_{y}^{2}$). Then $\bar{x}$ and $\bar{y}$ are effectively constant with respect to the changing population densities *P* and *N*_*i*_, *i* = 1,2. In which case, the population dynamics of the fast ecological system converges to a unique attractor with a time-averaged ecological state $\left(P^{*}(\bar{x},\bar{y}),N_{1}^{*}(\bar{x},\bar{y}),N_{2}^{*}(\bar{x},\bar{y})\right)$ equal to one of four equilibria (coexistence, predator exclusion, prey 1-exclusion, prey 2-exclusion) (Supporting Information Appendix D). Thus, on the evolutionary timescale, the trait dynamics are

(2) $$\frac{\text{d}\bar{x}}{\text{d}t}=\sigma_{x,G}^{2}\left[\left(b_{1}-m_{1}c_{1}{\bar{S}}_{1}(\bar{y})\right){\bar{a}}_{1}(\bar{x})N_{1}^{*}(\bar{x},\bar{y})\frac{\theta_{1}-\bar{x}}{\sigma_{x}^{2}+\zeta_{1}^{2}}\;\;+\left(b_{2}-m_{2}c_{2}{\bar{S}}_{2}(\bar{y})\right){\bar{a}}_{2}(\bar{x})N_{2}^{*}(\bar{x},\bar{y})\frac{\theta_{2}-\bar{x}}{\sigma_{x}^{2}+\zeta_{2}^{2}}\right],\;\frac{\text{d}\bar{y}}{\text{d}t}=\sigma_{y,G}^{2}\left[m_{1}c_{1}{\bar{a}}_{1}(\bar{x})N_{1}^{*}(\bar{x},\bar{y})\left(\beta_{1}-{\bar{S}}_{1}(\bar{y})\right)\frac{\phi_{1}-\bar{y}}{\sigma_{y}^{2}+\tau_{1}^{2}}\;\;+m_{2}c_{2}{\bar{a}}_{2}(\bar{x})N_{2}^{*}(\bar{x},\bar{y})\left(\beta_{2}-{\bar{S}}_{2}(\bar{y})\right)\frac{\phi_{2}-\bar{y}}{\sigma_{y}^{2}+\tau_{2}^{2}}\right].$$

For this reduced system (2), we calculate nullclines, stable and unstable equilibria, and separatrices across a range of foraging trade-offs.

Beyond the separation of timescale between ecological and evolutionary dynamics, the diet and immune traits can themselves evolve on different timescales. As the two traits are genetically independent, one trait may evolve on a slower timescale than the other if the two traits differ significantly in their genotypic variance or their selection pressure. These differences can arise in three ways, as discussed in the Results section. In particular, we consider the effects of (i) differences between the traits’ genotypic variances, (ii) rare parasites or parasites with weak effects on the predator, and (iii) differences between the strengths of the evolutionary trade-offs of the two traits.

### LATIN HYPERCUBE SAMPLING

**Figure 1.**
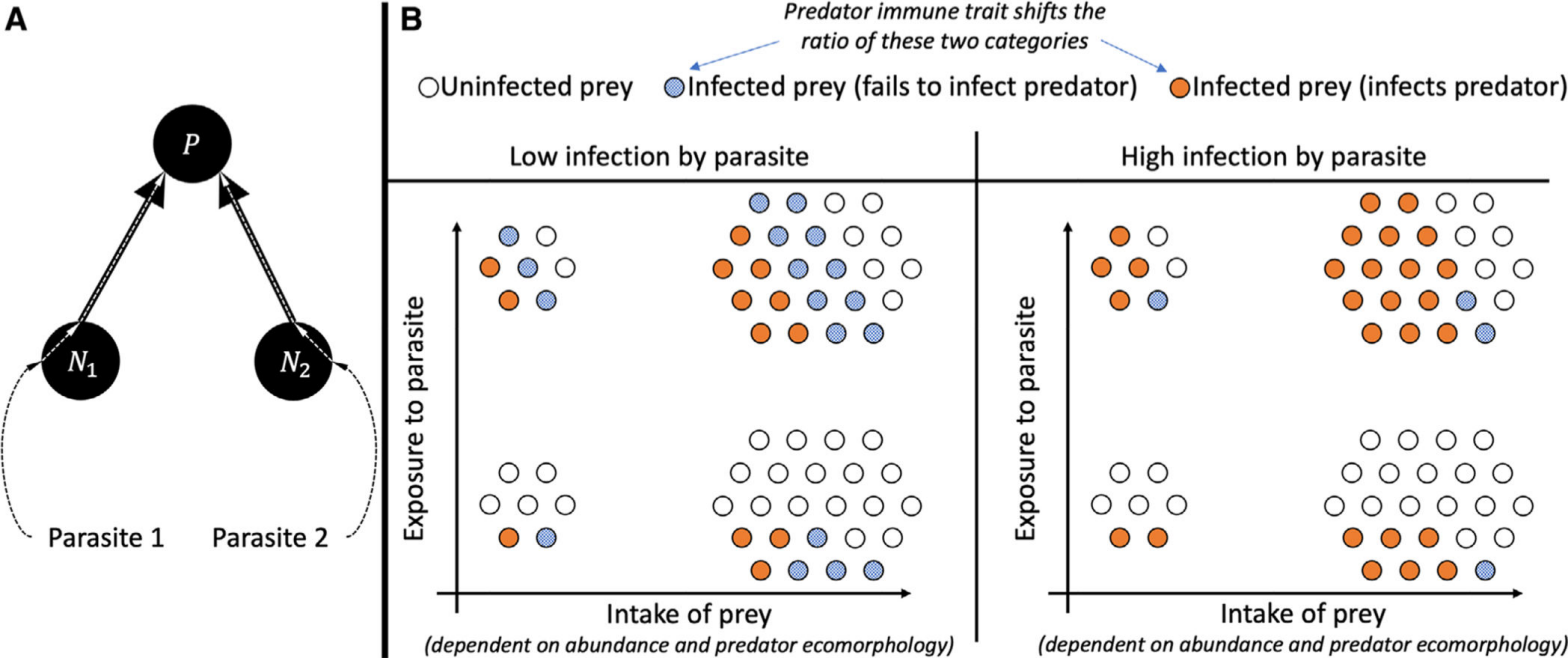
(A) Schematic of the model. The predator is exposed to limnetic and benthic parasites via intake of limnetic and benthic prey. The proportion of prey infected by parasites stays constant. The predator average morphology $\bar{x}$ and average immune response $\bar{y}$ evolves in response to selection pressures caused by prey availability and parasite infection. (B) Intake of prey *i* $\left({\bar{a}}_{i}(\bar{x})N_{i}\right)$ describes total intake of both infected and uninfected prey. Exposure to parasite i $\left({\bar{a}}_{i}N_{i}c_{i}\right)$ describes total intake of parasites (a constant proportion *c*_*i*_ of prey *i* areinfected with parasites). Infection by parasite *i* $\left({\bar{a}}_{i}N_{i}c_{i}{\bar{S}}_{i}(\bar{y})\right)$ describes the total number of ingested parasites that successfully infect thepredator (a proportion ${\bar{S}}_{i}(\bar{y})$ of ingested parasites infect the predator).

Using the equilibria of the slow-evolution model (2), we calculated the relative intake rates of the two prey types $\bar{a_{i}}(\bar{x})N_{i}/\sum_{k=1}^{2}{\bar{a}}_{k}(\bar{x})N_{k},\;(i=1,2)$, the relative exposure rates to the two parasite types $\bar{a_{i}}(\bar{x})N_{i}c_{i}/\sum_{k=1}^{2}\bar{a_{k}}(\bar{x})N_{k}c_{k},\;(i=1,2)$, as well as the relative parasite infection rates (Figure 1B) of the two parasite types ${\bar{a}}_{i}(\bar{x})N_{i}c_{i}{\bar{S}}_{i}(\bar{y})/\sum_{k=1}^{2}{\bar{a}}_{k}(\bar{x})N_{k}c_{k}{\bar{S}}_{k}(\bar{y}),(i=1,2)$ over a two-dimensional range of foraging trade-offs (ζ_*i*_) and immune trade-offs (τ_*i*_). For each trade-off pair, we ran 4000 simulations using Latin hypercube sampling, varying lake size (e.g., *K*_1_/*K*_2_), maximal attack rates (α_1_, α_2_), parasite frequency in prey (*c*_1_, *c*_2_), parasitic effects on stickleback (*m*_1_, *m*_2_), prey growth rates (*r*_1_, *r*_2_), and initial stickleback ecomorphology and immunity (${\bar{x}}_{0}$, ${\bar{y}}_{0}$). For each set of parameter values, we ran the two-timescale model (2) until $\bar{x}$ and $\bar{y}$ reached an evolutionary equilibrium and calculated the relative intake, exposure, and infection rates. We then plotted the results to gain insight about the joint evolution of diet and immune traits and how their evolution affects the relationship between diet, parasite exposure, and infection. A particular evolutionary equilibrium may not correspond to ecological coexistence. In Supporting Information Appendix G, we show that conditioning on coexistence does not alter our main conclusions.

To determine whether the correlation between diet and immune evolution (and, thus, between relative intake, exposure, and infection rates) is effected by dynamic proportions *c*_*i*_ or infected prey, we ran 4000 simulations of the seven-dimensional model described in Supporting Information Appendix F (dynamic *c*_*i*_). We used Latin hypercube sampling, varying parameters in an identical fashion to model (2), except for *c*_*i*_. The results are shown in Supporting Information Appendix F.

## Results

We first present results of the slow-evolution models to address how the evolution of diet affects the evolution of immunity and vice versa. We then present the Latin hypercube sampling results to address how the two traits are correlated across populations, as well as how that correlation affects the relationship between diet and infection across populations. We conclude with a “within populations” perspective using the full single-timescale five-dimensional model to examine temporal correlations in traits and diet and infection rates for systems with cyclic or chaotic dynamics.

### THREE-TIMESCALE DYNAMICS

For a given immune state $\bar{y}$, the average predator per-capita growth rate $\bar{W}$ (predator population per-capita growth rate) is unimodal with respect to the foraging trait $\bar{x}$ if

(3) $$\left|\theta_{1}-\theta_{2}\right|<2\sqrt{\sigma_{x}^{2}+\zeta^{2}},$$

where ζ := ζ_1_ = ζ_2_ (Schreiber et al. 2011; Schreiber and Patel 2015). Namely, if foraging trade-offs are weak relative to the phenotypic variation in foraging, then there is a single-predator population per-capita growth rate maximum with respect to *x*. When the contributions of the two prey populations to predator population per-capita growth rate are equal (i.e., $(b_{1}-c_{1}m_{1}{\bar{S}}_{1}(\bar{y}){\bar{a}}_{1}N_{1}=b_{2}-c_{2}m_{2}{\bar{S}}_{2}(\bar{y}){\bar{a}}_{2}N_{2}$), condition (3) is necessary and sufficient, but when prey contributions to predator fitness are unequal, predator population per-capita growth rate may be unimodal with respect to $\bar{x}$ even if (3) does not hold.

Similarly, for a given foraging trait $\bar{x}$, $\bar{W}$ is unimodal with respect to the immune trait $\bar{y}$ if the immune trade-off is weak relative to the phenotypic variance in immunity, that is,

(4) $$\left|\phi_{1}-\phi_{2}\right|<2\sqrt{\sigma_{y}^{2}+\tau^{2}},$$

where τ := τ_1_ = τ_2_ (Supporting Information Appendix E). This condition is necessary and sufficient only when the difference between the effects of parasites infecting predators maximally and minimally susceptible to those parasites is symmetric (i.e., $m_{1}c_{1}(\beta_{1}-\gamma_{1}){\bar{a}}_{1}(\bar{x})N_{1}=m_{2}c_{2}(\beta_{2}-\gamma_{2}){\bar{a}}_{2}(\bar{x})N_{2}$). Again, if these differences are unequal, $\bar{W}$ may still be unimodal with respect to $\bar{y}$ even if (4) does not hold.

**Figure 2.**
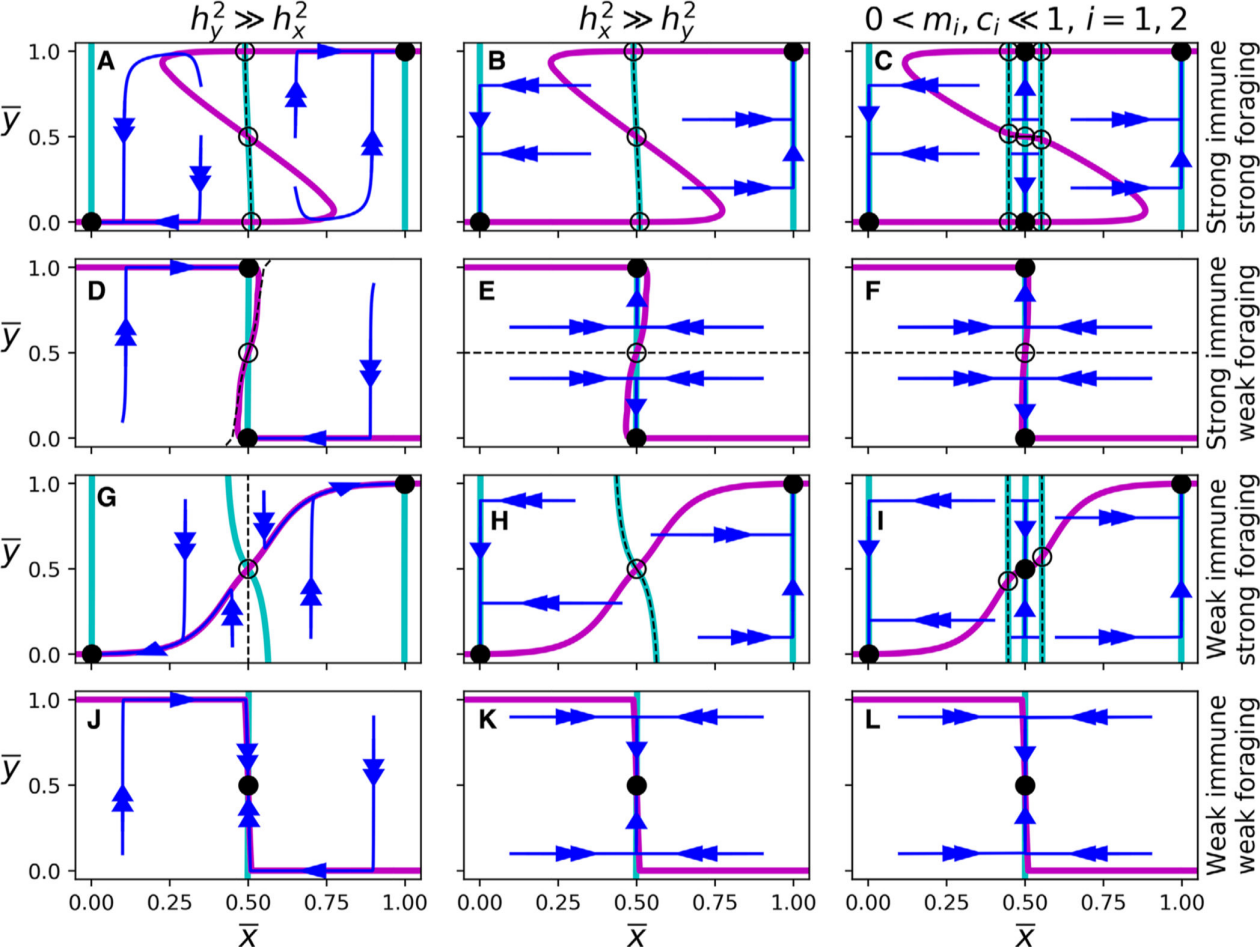
Nullclines, stable and unstable equilibria, separatrices, and evolutionary dynamics of Figure 2. The cyan and pink curves denote the $\bar{x}$- and $\bar{y}$-nullclines, respectively. Filled-in and hollow circles indicate stable and unstable evolutionary equilibria, respectively. Blue lines are sample trajectories, and dashed lines indicate separatrices between stable equilibria (as well as the stable manifolds of the saddles). In (A), (D), (G), and (J), σ_*x*,*G*_ = 0.005 and σ_*y*,*G*_ = 0.25. In (B), (E), (H), and (K), σ_*x*,*G*_ = 0.25 and σ_*y*,*G*_ = 0.005. In (C), (F), (I), and (L), *m*_*i*_ = *c*_*i*_ = 0.1 for *i* = 1,2. In (A)-(F) immune trade-offs are strong (τ_*i*_ = 0.01), and in (G)-(L) immune trade-offs are weak (τ_*i*_ = 1). In the (A)-(C) and (G)-(I) foraging trade-offs are strong (ζ_*i*_ = 0.01) and in (D)-(F) and (J)-(L) foraging trade-offs are weak (ζ_*i*_ = 1).

There are three ways in which the predator traits may evolve at different timescales. First, all else being equal, the trait with a higher genotypic variance evolves more quickly than the other (Figure 2A,B,D,E,G,H,J,K). Second, if the parasite is rare or has a weak effect on the predator, then selection pressure on the immune trait $\bar{y}$ is weak and therefore evolves much slower than the foraging trait $\bar{x}$ (Figure 2C,F,I,L). Third, weak trade-offs in either trait result in weak selection pressure on that trait. In particular, large ζ_*i*_ (the inverse strength of selection on $\bar{x}$) corresponds to slower $\bar{x}$ evolution (Figure 2D-F,J-L), and large τ_*i*_ (the inverse strength of selection on $\bar{y}$) corresponds to slower $\bar{y}$ evolution (Figure 2A-C,G-I).

Figure 2 shows the evolutionary dynamics of (2) for a variety of scenarios. It also highlights three major asymmetries of the foraging and immune traits in the context of three-timescale dynamics: (i) the relationship between trait trade-off and equilibrium location, (ii) the relationship between initial and final evolutionary state, and (iii) the directionality of trait evolution.

Predators evolve generalist foraging strategies if the foraging trade-off is weak, regardless of the immune trade-off (Figure 2DF,J-L). In contrast, predators evolve generalist immune strategies if the immune trade-off is weak, but only when the predator already has a generalist foraging strategy (Figure 2I-L). The immune trade-off needs to be very weak (in relation to the foraging trade-off) to have the same effect as the foraging trade-off.

The final foraging state is generally determined by the initial foraging state, regardless of the strengths of trait trade-offs. In contrast, the final immune state depends on both initial foraging and immune states, the strength of the trait trade-offs, and ecological parameters such as parasite prevalence and lethality. Graphically, the $\bar{x}$-nullclines in Figure 2 remain relatively vertical regardless of the strength of the foraging and immune trade-offs, in contrast to the $\bar{y}$-nullclines, which are never only horizontal. Consider, for example, a specialist predator (in both foraging and immune state) in an environment in which foraging and immune trade-offs are strong (Figure 2A-C). The stabilizing selection at this evolutionary state is strong enough to withstand weakening immune trade-offs, but not weakening foraging trade-offs. In fact, if the foraging trade-off becomes sufficiently weak, the predator will evolve a generalist foraging strategy, and an immune strategy dependent on the environment and the relative heritabilities of the two traits.

As a result of the extreme nature of the $\bar{x}$-nullclines, the foraging trait always evolves unidirectionally. On the other hand, because the $\bar{y}$-nullclines are not strictly horizontal, the immune state may reverse its evolution when immune heritability is high relative to foraging heritability (Figure 2A,G,J). In these scenarios, the immune state evolves quickly toward a stable branch of the $\bar{y}$-nullcline, and then both traits evolve along the $\bar{y}$-nullcline toward a stable evolutionary equilibrium.

### TWO-TIMESCALE DYNAMICS

**Figure 3.**
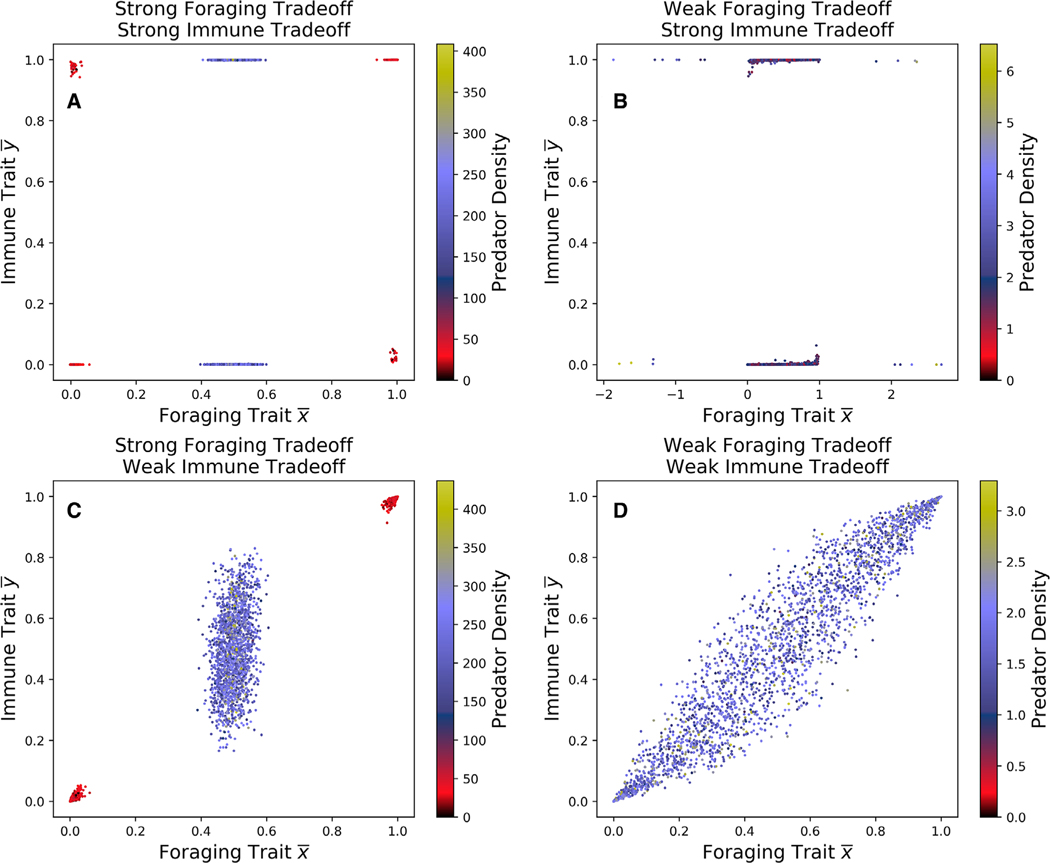
Locations of stable equilibria for a Latin hypercube sample of parameter space. In (A) and (B) immune trade-offs are strong (τ*i* = 0.01), and in (C) and (D) immune trade-offs are weak (τ*i* = 1). In (A) and (C) foraging trade-offs are strong (ζ*i* = 0.01) and in (B) and (D) foraging trade-offs are weak (ζ*i* = 1). The color of each dot represents the density of the predator population at the evolutionary equilibrium.

We used Latin hypercube sampling over a subset of parameter space to understand how the locations of the stable equilibria change as parameters vary (Figure 3). When both trade-offs are strong (Figure 3A), equilibria congregate near evolutionary specialist states, whereas when both trade-offs are weak (Figure 3D), the predator is more likely to evolve a generalist foraging and immune strategy. If foraging trade-offs are weak and immune trade-offs are strong (Figure 3B), predators will typically evolve a generalist foraging strategy and a specialist immune strategy. In contrast, if immune trade-offs are weak and foraging trade-offs are strong (Figure 3C), then predators may evolve a generalist or specialist foraging strategy, and the immune strategy will evolve to correspond with the foraging state. We see the same asymmetry as in Figure 2: generalist immune traits only evolve for generalist foragers, but generalist foraging traits may evolve regardless of immune state.

**Figure 4.**
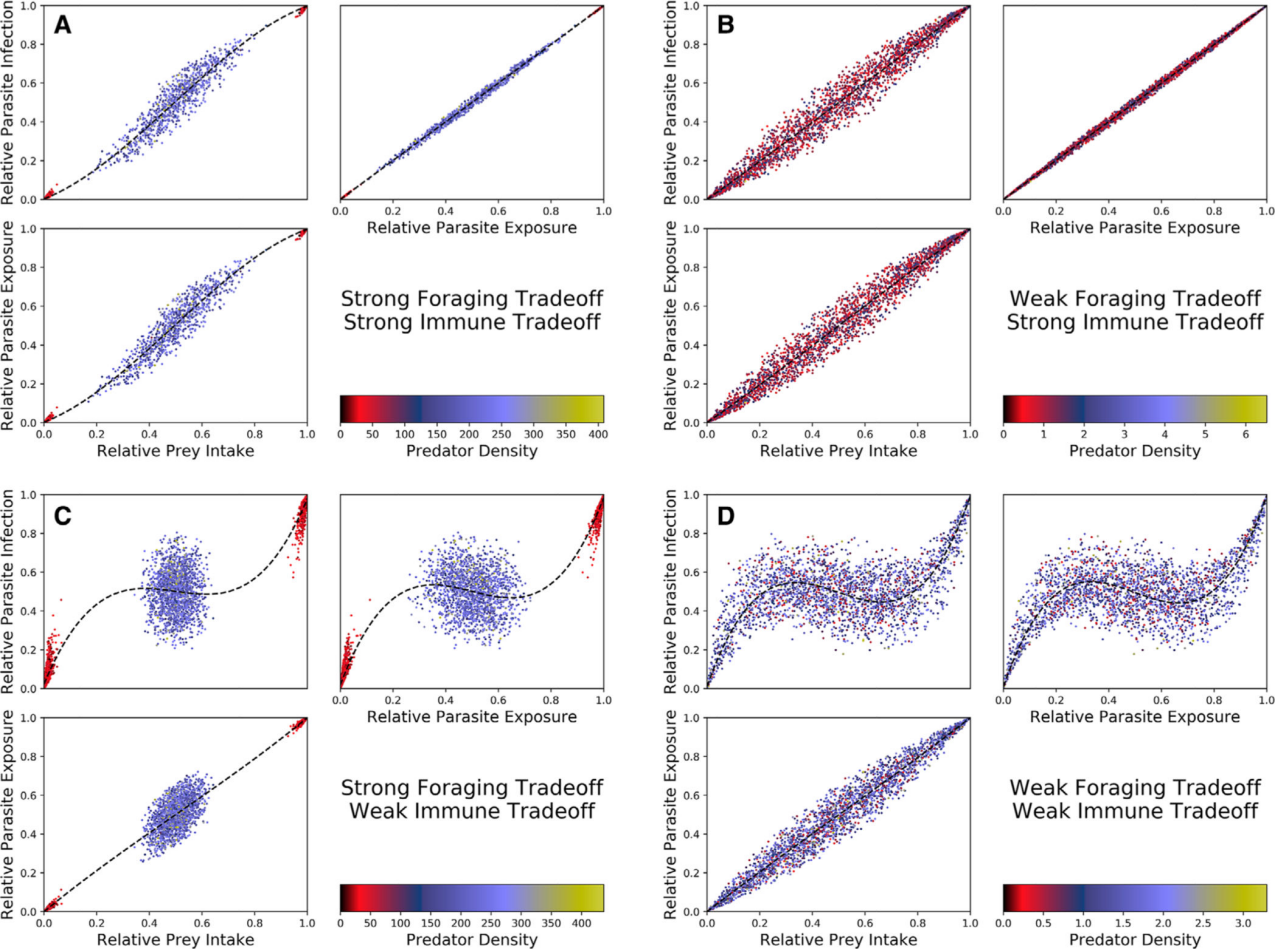
Prey intake, parasite exposure, and parasite infection over the same subset of parameter space given in Figure 3. The dots are colored as in Figure 3. The blue lines are splines of the data, included to better identify patterns between prey intake, parasite exposure, and parasite infection. In (A) and (B) immune trade-offs are strong (τ*i* = 0.01), and in (C) and (D) immune trade-offs are weak (τ*i* = 1). In (A) and (C), foraging trade-offs are strong (ζ*i* = 0.01) and in (B) and (D) foraging trade-offs are weak (ζ*i* = 1).

We also used the same Latin hypercube sample to understand what determines correlations between prey intake, parasite exposure, parasite infection across predator populations (Figure 4). When immune trade-offs are strong (Figure 4A,B), the relationship between prey intake and parasite infection does not stray far from the one-to-one line. In these scenarios, the proportion of a predator population’s diet consisting of some prey is roughly equal to the proportion of that predator’s parasite load consisting of the parasites from that prey. In addition, the majority of the variation in parasite infection is caused by the relationship between prey intake and parasite exposure, and not between exposure and infection. This means that any potential nonlinear pattern between diet and infection is not caused by immune evolution if immune trade-offs are strong.

When immune trade-offs are weak (Figure 4C,D), the relationship between prey intake and parasite infection differs greatly from the one-to-one line. In these scenarios, the proportion of a predator population’s diet consisting of some prey may not predict the proportion of that predator population’s parasite load consisting of the parasites from that prey. In contrast to when immune trade-offs are strong, the majority of the variation in parasite infection *is* caused by the relationship between parasite exposure and parasite infection, indicating that any potential nonlinear pattern between diet and infection is caused by immune evolution if immune trade-offs are weak.

### NONEQUILIBRIUM ECOEVOLUTIONARY DYNAMICS

**Figure 5.**
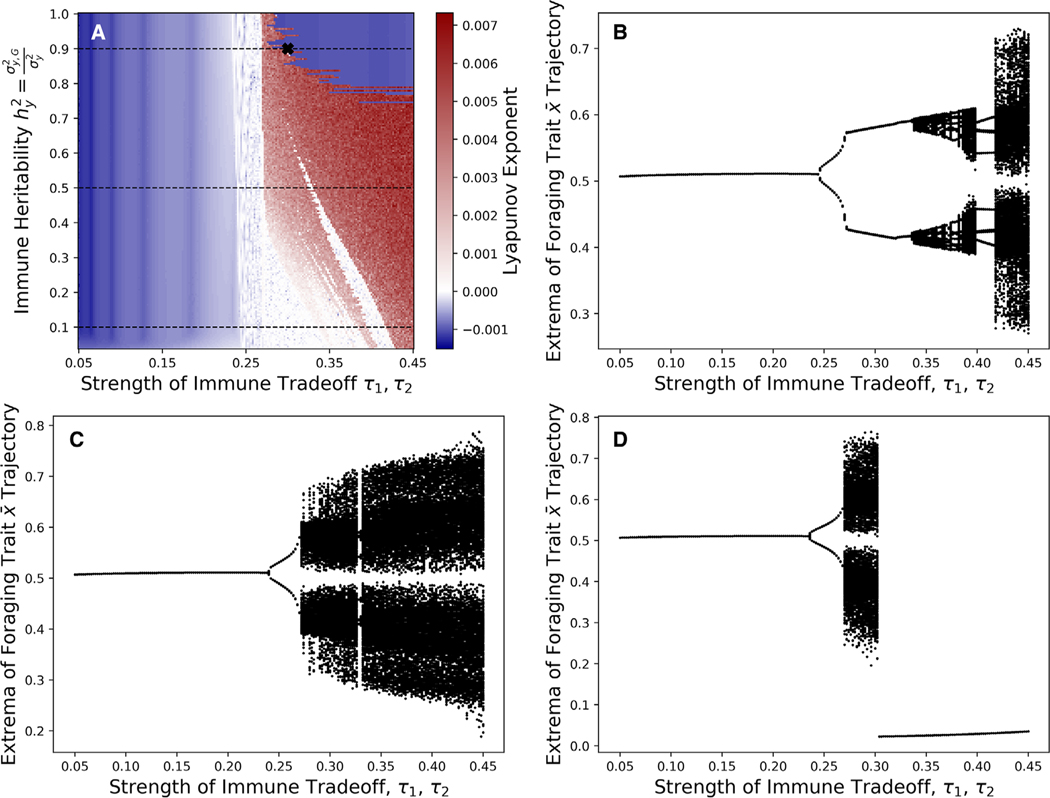
When immune trade-offs are sufficiently weak, immune evolution can induce cyclic or chaotic ecoevolutionary dynamics. For weak immune trade-offs, low heritability is destabilizing, but for intermediate immune trade-offs, high heritability is destabilizing. (A) The blue regions denote stability (negative Lyapunov exponent) and the red regions denote chaos (positive Lyapunov exponent). (B) Local extrema of the diet trait $\bar{x}$ along 0.05 ≤ τ_1_ = τ_2_ ≤ 0.45, $h_{y}^{2}$ = 0.1. (C) Local extrema of the diet trait *x* along 0.05 ≤ τ_1_ = τ_2_ ≤ 0.45, $h_{y}^{2}$ = 0.5. (D) Local extrema of the diet trait *x* along 0.05 ≤ τ_1_ = τ_2_ ≤ 0.45, $h_{y}^{2}$ = 0.9.

When heritability is high, ecoevolutionary feedbacks lead to greater dynamical complexity, including cyclical and chaotic dynamics. Schreiber et al. (2011) showed diet evolution can induce cycles and chaos in the absense of immune evolution, and we found something similar for immune evolution. Regardless of immune heritability, ecoevolutionary cycles only occur for sufficiently weak immune trade-offs (Figure 5A). When immune heritability is low (Figure 5B), chaos occurs for very weak immune trade-offs, whereas for higher immune heritability (Figure 5C,D), chaos occurs for intermediate and possible also very weak immune trade-offs.

**Figure 6.**
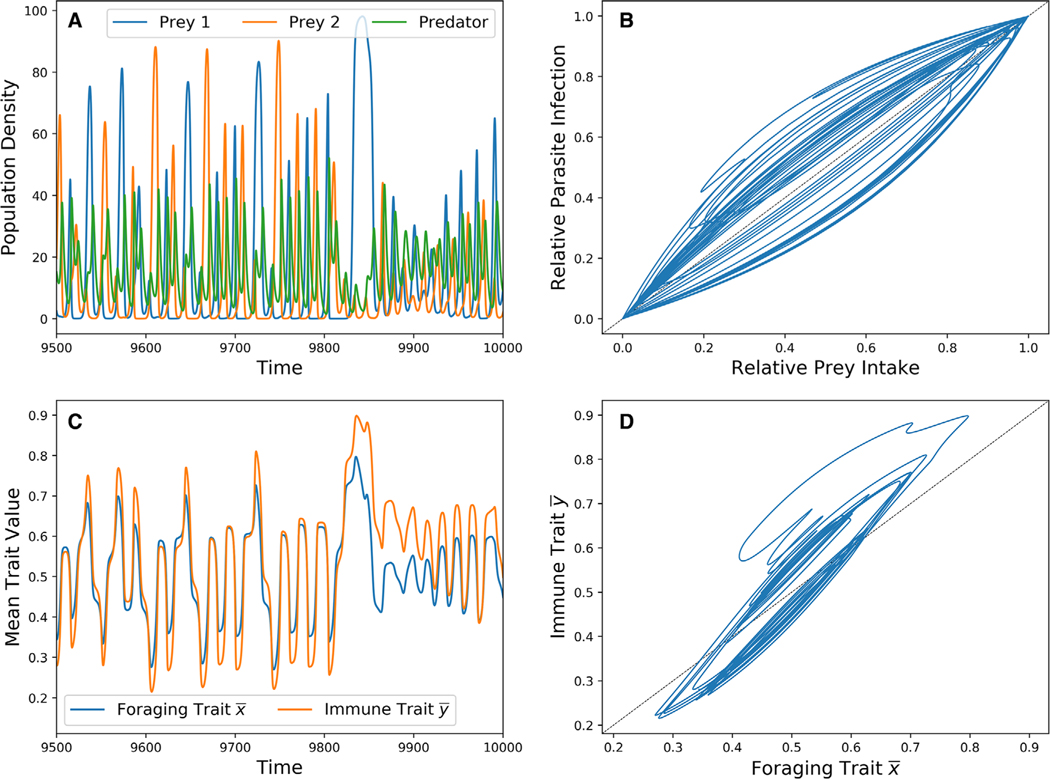
A chaotic ecoevolutionary trajectory. Parameters are equal to that of Figure 5 with τ_1_ = τ_2_ = 0.3 and $h_{y}^{2}$ = 0.9 (bold x in Figure 5A). (A) Predator and prey densities through time. (B) Predator relative prey intake versus relative parasite infection. (C) Predator foraging and immune traits through time. (D) Predator foraging trait versus immune trait.

A typical chaotic ecoevolutionary trajectory is displayed in Figure 6. These dynamics show a positive temporal correlation between foraging and immune traits (Figure 6C,D). When the predator foraging trait favors one prey type over the other, its intake almost entirely consists of that prey. The immune trait has higher heritability than the foraging trait, which is why the immune trait evolves more extreme values than the foraging trait. Once the predator overconsumes a particular prey type and the other recovers, the foraging trait faces directional selection toward the recovering, although there is a lag in the actual intake of that prey. Although it is highly heritable, the immune trait does not favor the parasite of the recovering prey until the foraging trait is near its extreme value.

There is also a nonlinear correlation between diet and infection (Figure 6B). Because this correlation occurs within a single-oscillating population, the intake-exposure relationship is one to one. Thus, any nonlinear correlation between diet and infection is entirely caused by the evolving immune trait $\bar{y}$.

## Discussion

In this study we addressed three questions: (a) how the evolution of a predator’s diet affects evolution of immunity to trophically transmitted parasites, and vice versa; (b) how these traits are correlated across populations and within a single population undergoing ecoevolutionary oscillations; and (c) how the simultaneous evolution of diet and immunity can affect the correlation between intake of and infection by parasites among populations and within a single population undergoing ecoevolutionary oscillations.

For (a), we found that even when the predator’s immune state evolves faster than its diet (due to high immune heritability and low ecomorphology heritability), immune evolution does not determine diet. On the other hand, diet evolution often determines the predator’s immune state, regardless of the relative speeds of evolution. Indeed, a fast-evolving immune state may reverse its evolution in response to a shifting diet, but the diet does not respond to a shifting immunity.

The asymmetry between diet and immunity extends beyond the question of which trait determines the other. When diet trade-offs are weak and immune trade-offs are strong, the predator maintains a generalist foraging ecomorphology even though its immunity is specialized against one parasite or the other. This is true even if the parasites are abundant and detrimental to the predator’s fitness. On the other hand, when diet trade-offs are strong and immune trade-offs are weak, the predator evolves a specialist foraging ecomorphology along with a specialist immunity against the parasites it encounters. In short, there is little fitness benefit in maintaining an immunity to a parasite rarely encountered, but there is significant benefit to maintaining a morphology suitable to consume multiple prey even when they contain parasites that confer significant fitness drawbacks. This theoretical result runs contrary to suggestions that parasite infection risk drove the evolution of a narrower diet in Tropheini cichlids (Hablützel et al. 2017).

When ecoevolutionary dynamics occur on commensurate timescales, our numerical results suggest that oscillations do not occur when immunological trade-offs are sufficiently strong. Theory predicts evolutionary destabilization occurs more commonly when there is a trade-off between capturing different prey phenotypes (Abrams and Matsuda 1997a, 1997b; Abrams 2000), but we find that there is a limit to this effect; if trade-offs are too strong, evolutionary oscillations are suppressed.

For (b) and (c), our Latin hypercube sampling results showed no correlation between trophic and immune traits when immune trade-offs are strong. With these strong trade-offs, predators always evolve a specialized immunity regardless of their ecomorphology. In contrast, when immune trade-offs are weak, predators evolve an immunity to suit their diet. Here, the strength of foraging trade-offs determine the correlation; if foraging trade-offs are strong, then predators evolve to only a few morphological states (and thus only a few immunological states), whereas if foraging trade-offs are weak, then predators may evolve anywhere along the ecomorphology spectrum (and thus anywhere along the immunological spectrum).

Because predators only evolve immunity to suit their diet if immune trade-offs are weak, negative correlations between parasite intake and infection are only possible with weak immune trade-offs. The negative correlation between intake and infection is more pronounced when there is more morphological diversity across populations, and thus is most likely to be observed if foraging trade-offs are also weak. These results, when compared, to the empirical data of Stutz et al. (2014), suggest that evolutionary trade-offs in stickleback diet (benthic or limnetic prey) and immune traits (benthic or limnetic parasite) are likely to be weak, which aligns with other studies that have shown that many stickleback populations have evolved a generalist morphology when both limnetic and benthic prey are present (Lavin and McPhail 1985; Schluter and McPhail 1992; Matthews et al. 2010; Snowberg et al. 2015).

We also found a nonlinear correlation between intake and infection within a single population oscillating in time. Because of the assumption that parasite abundance stays constant, this correlation is caused entirely by the evolution of immunity. Our simulations did not produce a negative correlation between intake and infection within a single-oscillating population. Nevertheless, the nonlinear correlation suggests that the negative correlation between diet and infection across populations observed by Stutz et al. (2014) may have resulted from oscillating populations in similar habitats rather than equilibrated populations in different habitats. However, because ecological variation among lakes is correlated with lake size (larger lakes containing more limnetic-feeding populations, Bolnick and Ballare 2020), this is unlikely to be the case.

It is well known that stickleback face biomechanical trade-offs that limit their ability to capture both benthic and limnetic prey (Robinson 2000). In contrast, it is not known whether stickleback immunity faces comparable immunological trade-offs. That is, does immunity to benthic-derived parasites (e.g., nematodes) also confer protection to limnetic-derived parasites (e.g., *Schistocephalus* cestodes), or inhibit immunity to cestodes? In general, evidence suggests that different parasites are detected by different host MHC IIb alleles (Stutz and Bolnick 2017), suggesting a possible trade-off. For certain kinds of parasites this trade-off is well documented, such as the mutual inhibition of Th1 and Th2 adaptive immune responses that, respectively, target bacterial and helminth infections in mammals such as Cape Buffalo (Ezenwa et al. 2010). We chose to model stickleback immunity on a single bidirectional axis, with different optimal values for immunity against limnetic and benthic parasites. This choice comes with the implicit assumption that there is a limited amount of energy allocated toward immunity. However, the vertebrate immune system is complex and highly multivariate, and there exist alternative means of modeling immunity to different parasites. Understanding how these alternative approaches (e.g., few loci of large effect vs. a multivariate quantitative trait) infuences eco-evo-immunological dynamics is an important challenge for future work.

This study was motivated in part by the specific relationship between stickleback and their infected prey. However, trophically transmitted parasites are very common in nature (Combes 2001). This study helps shed light on how food web dynamics are affected by the presence of diet-derived parasites, and is a contribution to the growing body of theory regarding eco-evo dynamics in a multispecies context (Saloniemi 1993; Abrams and Matsuda 1997b; Abrams 2006; Schreiber et al. 2011; Vasseur and Fox 2011; Tien and Ellner 2012; Cortez and Weitz 2014; Patel and Schreiber 2015; Schreiber and Patel 2015; Klauschies et al. 2016; Cortez and Patel 2017; Fleischer et al. 2018; Patel and Schreiber 2018; Patel and Bürger 2019; Cortez et al. 2020). Conversely, we need to improve our understanding of how food web dynamics play a role in the dynamics of trophically transmitted parasites, and future theoretical studies should incorporate the dynamics of parasites along with the dynamics of the community in which they reside. Little is known, for example, about the dual effects of parasite dynamics and predator evolution on coexistence of a predator with two competing prey. Prosnier et al. (2020) used an epidemiological framework to examine the effect of prey infection on predator diet. They also examined the effect of predator diet evolution on coexistence using an adaptive dynamics evolutionary framework and showed that this type of evolution generally promotes coexistence among a predator and an infected and uninfected prey. Like in this study, reductions in prey density correspond to lower consumption rates by the predator, which ultimately favors prey persistence.

Prosnier et al. (2020) modeled parasite transmission as horizontal between prey. This is not the case for stickleback prey, which become infected by parasites through consumption (Barber and Scharsack 2009). We therefore chose not to include explicit parasite dynamics or the epidemiological dynamics of the prey, and instead assumed that the proportion of prey which are infected stays constant. Common predators of stickleback are piscivorous birds which freely move between many lakes or ponds. Parasites lay eggs in the gut of a bird and these eggs are deposited into lakes when these birds defecate above water. Genetic evidence suggests that a significant proportion of the parasite load in prey results from regional recruitment rather than local population reproduction (Shim and Bolnick, unpubl. ms.). That is, birds that consume infected stickleback from one lake or pond often defecate into another, which keeps the parasite load in each lake or pond relatively constant.

This is not necessarily the case in all scenarios where there are multiple prey species hosting different trophically transmitted parasites. To understand model sensitivity to this assumption, we formulated and analyzed a model in which the proportion *c*_*i*_ of infected prey is a dynamic variable dependent on predator consumption of infected prey (Supporting Information Appendix F). Our analysis suggests that many of the main results hold even when parasite recruitment is mostly local. However, there are two key differences that warrant further investigation. First, there are instances in which the predator evolves a generalist immune trait despite specializing its morphology, which is not seen when parasite infection rates are constant. Second, dynamic parasite infection rates appears to strengthen the relationship between prey intake and parasite exposure, suggesting that evolution of predator immunity determines correlations between prey intake and parasite infection, regardless of the strength of the morphological and immunological trade-offs.

Although it may be that immunity and ecomorphology are genetically linked in some way, we chose to model the two traits as genetically independent. This choice improves mathematical tractability, as well as provides an example of a system in which the evolution of two traits drive each other, not because of genetic linkage, but rather based solely on interdependent selection pressures. Future studies should explore how the interaction between correlated selection pressures and genetic linkage affects the applicability of our results.

Finally, experiments are needed to validate our model, including measurements of relevant parameters and tests of our assumptions. In the case of stickleback, we know that individuals vary in their propensity to consume benthic versus limnetic resources (Robinson 2000; Bolnick and Lau 2008; Matthews et al. 2010; Bolnick et al. 2014; Snowberg et al. 2015). However, the precise nature and strength of the biomechanical (and perhaps cognitive) trade-offs remain poorly understood (Robinson 2000; Schmid et al. 2019). Likewise, we know that stickleback genotypes differ in their resistance to various parasites (Kalbe and Kurtz 2005; MacColl 2009; MacColl and Chapman 2010; Eizaguirre et al. 2012; Nagar and MacColl 2016; Stutz and Bolnick 2017; Weber et al. 2017a, 2017b, among many others). But, we know little about trade-offs (or synergy) between resistance to different parasites. For that matter, parasites can manipulate host immunity in ways that benefits or harms co-infecting parasites (e.g., Ezenwa et al. 2010). We therefore need to bring together biomechanical studies of foraging trade-offs, with mechanistic immunological studies of resistance trade-offs. In addition, we lack sufficient information about the relative virulence of different parasites acquired through alternative prey, and future theoretical studies should include the effects and evolution of all three host strategies: avoidance, resistance, and tolerance.

## Supplementary Material

Appendix A **Table B1**: Baseline parameter values.
**Table B2**: Figure 2 parameters. All parameters not given here are given in Table B1.
**Table B3**: Figures 3 and 4 parameters. All parameters not given here are given in Table B1.
**Figure F1**: Locations of Stable equilibria for a Latin Hypercube sample of parameter space for *l*1 = *l*2 = 1.
**Figure F2**: Prey intake, parasite exposure, and parasite infection over the same subset of parameter space given in Figure F1.
**Figure F3**: Locations of Stable equilibria for a Latin Hypercube sample of parameter space for *l*1 = *l*2 = 10.
**Figure F4**: Prey intake, parasite exposure, and parasite infection over the same subset of parameter space given in Figure F3.
**Figure F5**: Locations of Stable equilibria for a Latin Hypercube sample of parameter space for *l*1 = *l*2 = 100.
**Figure F6**: Prey intake, parasite exposure, and parasite infection over the same subset of parameter space given in Figure F5.
**Table 1**: The numbers of simulations which result in noncoexistence in each of the four scenarios (weak and strong foraging and immune trade-offs).
**Figure G1**: Prey intake, parasite exposure, and parasite infection over a subset of parameter space, conditional on all three species coexisting.
**Figure G2**: The equilibrium conditions of $\frac{r1}{\bar{a}1}$ and $\frac{r2}{\bar{a}2}$ over a subset of parameter space, conditional on one species being excluded.

Supplementary Figure 2

Supplementary Figure 1

Supplementary Figure 3

Supplementary Figure 4

Supplementary Figure 6

Supplementary Figure 8

Supplementary Figure 5

Supplementary Figure 7

Supplementary Figure 9

Tex File 2

Tex File 1

Tex File 3

Tex File 5

Tex File 4

Tex File 6

Tex File 8

Tex File 7

Bib File

## Figures and Tables

**Table 1.** Model symbols and descriptions.

Symbol	Description
*N*_*i*_	Prey *i* density, *i* = 1,2.
P	Predator density.
$\bar{x}$	Mean morphology trait value for predator population.
$\bar{y}$	Mean immune trait value for predator population.
*a*_*i*_(*x*)	Attack rate of a predator with morphology *x* on prey *i*.
α_*i*_	Maximum attack rate on prey *i*.
θ_*i*_	The value of *x* for which *a*_*i*_(*x*) is maximized.
ζ_*i*_	The “width” of the attack rate curve *a*_*i*_(*x*). Smaller values result in greater reduction in attack rate due to suboptimal morphology.
${\bar{a}}_{i}(\bar{x})$	Mean attack rate of predator population on prey *i*.
*S*_*i*_(*y*)	Susceptibility of a predator with immunity trait *y* to parasite *i*.
β_*i*_	Maximum predator susceptibility to parasite *i* infection.
γ_*i*_	Minimum predator susceptibility to infection by parasite *i*.
φ_*i*_	The value of *y* for which *S*_*i*_(*y*) is minimized.
τ_*i*_	The “width” of the susceptibility curve *S*_*i*_(*y*). Smaller values result in greater increase in susceptibility due to suboptimal immunology.
${\bar{S}}_{i}(\bar{y})$	Mean susceptibility of predator population to parasite *i*.
$\sigma_{x}^{2},\sigma_{y}^{2}$	Phenotypic variances of the morphological and immunological traits, *x* and *y*.
$\sigma_{x,G}^{2},\sigma_{x,E}^{2}$	Genetic and environmental components of the predator morphological trait variance.
$\sigma_{y,G}^{2},\sigma_{y,E}^{2}$	Genetic and environmental components of the predator immunological trait variance.
$r_{i},K_{i}$	Intrinsic growth rates and carrying capacities of prey *i*, *i* = 1,2.
$b_{i},d$	Predator conversion efficiency of prey *i*, and the intrinsic predator per-capita death rate.
$m_{i}$	Predator mortality induced by the infection of parasite *i*.
$c_{i}$	The proportion of prey *i* infected with parasite *i*.
