# Supplementary material for: Sick of eating: Eco‐evo‐immuno dynamics of predators and their trophically acquired parasites
Source: Evolution. 2021 Oct 12;75(11):2842–56. doi: 10.1111/evo.14353 (PMC8985590; doi:10.1111/evo.14353)
Supplement: Supplementary file 1 — Table B1: Baseline parameter values. Table B2: Figure 2 parameters. All parameters not given here are given in Table B1. Table B3: Figures 3 and 4 parameters. All parameters not given here are given in Table B1. Figure F1: Locations of Stable equilibria for a Latin Hypercube sample of parameter space for l1=l2=1. Figure F2: Prey intake, parasite exposure, and parasite infection over the same subset of parameter space given in Figure F1. Figure F3: Locations of Stable equilibria for a Latin Hypercube sample of parameter space for l1=l2=10. Figure F4: Prey intake, parasite exposure, and parasite infection over the same subset of parameter space given in Figure F3. Figure F5: Locations of Stable equilibria for a Latin Hypercube sample of parameter space for l1=l2=100. Figure F6: Prey intake, parasite exposure, and parasite infection over the same subset of parameter space given in Figure F5. Table 1: The numbers of simulations which result in noncoexistence in each of the four scenarios (weak and strong foraging and immune trade‐offs). Figure G1: Prey intake, parasite exposure, and parasite infection over a subset of parameter space, conditional on all three species coexisting. Figure G2: The equilibrium conditions of r1a¯1 and r2a¯2 over a subset of parameter space, conditional on one species being excluded. [file EVO-75-2842-s001.zip › evo14353-sup-0001-SuppMat.pdf]

Appendix A: Derivation of average prey and predator fitness

Recall the probability distributions $p_x(x, \overline{x})$ and $p_y(y, \overline{y})$:

$$p_x(x,\overline{x}) = \frac{1}{\sqrt{2\pi\sigma_x^2}} \exp\left[-\frac{(x-\overline{x})^2}{2\sigma_x^2}\right] \qquad \text{and} \qquad p_y(y,\overline{y}) = \frac{1}{\sqrt{2\pi\sigma_y^2}} \exp\left[-\frac{(y-\overline{y})^2}{2\sigma_y^2}\right] \quad \text{(A1)}$$

Given $W(x, y) = \sum_{i=1}^{2} \Big[(b_i - c_i m_i S_i(y))\, a_i(x) N_i \Big] - d$, then through the linearity of sums and integrals, we have

$$\overline{W}(\overline{x},\overline{y}) = \int_{\mathbb{R}\times\mathbb{R}} W(x,y) p_x(x,\overline{x}) p_y(y,\overline{y}) \mathrm{d}x\mathrm{d}y = \sum_{i=1}^{2} \Big[(b_i - c_i m_i \overline{S}_i(\overline{y})) \overline{a}_i(\overline{x}) N_i \Big] - d \quad \text{(A2)}$$

where

$$\overline{a}_i(\overline{x}) = \int_{\mathbb{R}} a_i(x) p_x(x,\overline{x}) \mathrm{d}x \qquad \text{and} \qquad \overline{S}_i(\overline{y}) = \int_{\mathbb{R}} S_i(y) p_y(y,\overline{y}) \mathrm{d}y \quad \text{(A3)}$$

are the average attack rate of the predator on prey i and the susceptibility rate of the predator to infection by the parasite in prey i, respectively. Similary, given $Y_i(x) = r_i \left(1 - \frac{N_i}{K_i}\right) - a_i(x)P$ for $i = 1, 2$, we have

$$\overline{Y}_i(\overline{x}) = \int_{\mathbb{R}} Y_i(x) p_x(x,\overline{x}) \mathrm{d}x = r_i \left(1 - \frac{N_i}{K_i}\right) - \overline{a}_i(\overline{x}) P. \quad \text{(A4)}$$

Finally, since

$$a_i(x) = \alpha_i \exp\left[-\frac{(x-\theta_i)^2}{2\zeta_i^2}\right] \qquad \text{and} \qquad S_i(y) = \beta_i - (\beta_i - \gamma_i)\exp\left[-\frac{(y-\phi_i)^2}{2\tau_i^2}\right], \tag{A5}$$

we have

$$\overline{a}_i(\overline{x}) = \int_{\mathbb{R}} \alpha_i \exp\left[-\frac{(x-\theta_i)^2}{2\zeta_i^2}\right] \frac{1}{\sqrt{2\pi\sigma_x^2}} \exp\left[-\frac{(x-\overline{x})^2}{2\sigma_x^2}\right] \mathrm{d}x \tag{A6}$$

$$= \frac{\alpha_i}{\sqrt{2\pi\sigma_x^2}} \int_{\mathbb{R}} \exp\left[-\frac{(x-\theta_i)^2}{2\zeta_i^2} - \frac{(x-\overline{x})^2}{2\sigma_x^2}\right] \mathrm{d}x \tag{A7}$$

$$= \frac{\alpha_i}{\sqrt{2\pi\sigma_x^2}} \int_{\mathbb{R}} \exp\Big[-\frac{1}{2\zeta_i^2\sigma_x^2}\big((\sigma_x^2+\zeta_i^2)x^2 - (2\theta_i\sigma_x^2 + 2\overline{x}\zeta_i^2)x + (\theta_i^2\sigma_x^2 + \overline{x}^2\zeta_i^2)\big)\Big]\mathrm{d}x \tag{A8}$$

$$= \frac{\alpha_i}{\sqrt{2\pi\sigma_x^2}} \frac{\sqrt{\pi}}{\sqrt{A}} \exp\left[\frac{B^2}{4A} - C\right] \tag{A9}$$

where $A = \dfrac{\sigma_x^2+\zeta_i^2}{2\zeta_i^2\sigma_x^2}$, $B = \dfrac{2\theta_i\sigma_x^2 + 2\overline{x}\zeta_i^2}{2\zeta_i^2\sigma_x^2}$, and $C = \dfrac{\theta_i^2\sigma_x^2 + \overline{x}^2\zeta_i^2}{2\zeta_i^2\sigma_x^2}$. So,

$$\frac{B^2}{4A} - C = -\frac{(\overline{x}-\theta_i)^2}{2\left(\sigma_x^2+\zeta_i^2\right)} \tag{A10}$$

and thus

$$\overline{a}_i(\overline{x}) = \frac{\alpha_i\zeta_i}{\sqrt{\sigma_x^2+\zeta_i^2}} \exp\left[-\frac{(\overline{x}-\theta_i)^2}{2\left(\sigma_x^2+\zeta_i^2\right)}\right]. \tag{A11}$$

Also,

$$\overline{S}_i\left(\overline{y}\right) = \int_{\mathbb{R}} \left(\beta_i - (\beta_i-\gamma_i)\exp\left[-\frac{y-\phi_i}{2\tau_i^2}\right]\right) \frac{1}{\sqrt{2\pi\sigma_y^2}} \exp\left[-\frac{(y-\overline{y})^2}{2\sigma_y^2}\right] \mathrm{d}y \tag{A12}$$

$$= \beta_i \int_{\mathbb{R}} \frac{1}{\sqrt{2\pi\sigma_y^2}} \exp\left[-\frac{(y-\overline{y})^2}{2\sigma_y^2}\right] \mathrm{d}y - \frac{\beta_i-\gamma_i}{\sqrt{2\pi\sigma_y^2}} \int_{\mathbb{R}} \exp\left[-\frac{(y-\phi_i)^2}{2\tau_i^2} - \frac{(y-\overline{y})^2}{2\sigma_y^2}\right] \mathrm{d}y \tag{A13}$$

Through an identical calculation as for $\overline{a}_i(\overline{x})$, we have

$$\overline{S}_i\left(\overline{y}\right) = \beta_i - \frac{(\beta_i - \gamma_i)\tau_i}{\sqrt{\sigma_y^2 + \tau_i^2}} \exp\left[-\frac{(\overline{y} - \phi_i)^2}{2\left(\sigma_y^2 + \tau_i^2\right)}\right]. \tag{A14}$$

Appendix B: Model parameters

Parameter	Baseline Value
K_1, K_2	100
α_1, α_2	0.7
β_1, β_2	0.95
γ_1, γ_2	0.05
σ_x, σ_y	0.25
$\sigma_{x,G}$, $\sigma_{y,G}$	0.1
τ_1, τ_2	0.1
ζ_1, ζ_2	0.1
θ_1, ϕ_1	0
θ_2, ϕ_2	1
b_1, b_2	1
c_1, c_2	0.9
m_1, m_2	0.9
r_1, r_2	1
d	0.4

Table B1: Baseline parameter values.

Figure(s)	Parameter	Value
Figure 2a-c,g-i	ζ_1, ζ_2	0.01
Figure 2d-f,j-l	ζ_1, ζ_2	1
Figure 2a-f	τ_1, τ_2	0.01
Figure 2g-l	τ_1, τ_2	1
Figure 2a,d,g,j	$\sigma_{x,G}$	0.005
Figure 2a,d,g,j	$\sigma_{y,G}$	0.25
Figure 2b,e,h,k	$\sigma_{x,G}$	0.25
Figure 2b,e,h,k	$\sigma_{y,G}$	0.005
Figure 2c,f,i,l	c_1, c_2, m_1, m_2	0.1

Table B2: Figure 2 parameters. All parameters not given here are given in Table B1.

Figure(s)	Parameter	Value
Figures 3a-d and 4a-d	K_1, K_2	$[10, 1000]$
Figures 3a-d and 4a-d	α_1, α_2	$[0.5, 0.9]$
Figures 3a-d and 4a-d	β_1, β_2	$[0.9, 1]$
Figures 3a-d and 4a-d	γ_1, γ_2	$[0, 0.1]$
Figures 3a-d and 4a-d	$\sigma_{x,G}$, $\sigma_{y,G}$	0.25
Figures 3a-d and 4a-d	b_1, b_2	$[0.8, 1.2]$
Figures 3a-d and 4a-d	c_1, c_2, m_1, m_2	$[0.5, 1]$
Figures 3a-d and 4a-d	r_1, r_2	$[0.5, 1.5]$
Figures 3a-d and 4a-d	d	$[0.25, 0.55]$
Figures 3a,b and 4a,b	τ_1, τ_2	0.01
Figures 3c,d and 4c,d	τ_1, τ_2	1
Figures 3a,c and 4a,c	ζ_1, ζ_2	0.01
Figures 3b,d and 4b,d	ζ_1, ζ_2	1

Table B3: Figures 3 and 4 parameters. All parameters not given here are given in Table B1.

Appendix C: Numerical approximation of Lyapunov exponents

Consider a system of ordinary differential equations

$$\dot{\mathbf{x}} = f(\mathbf{x}) \tag{C1}$$

and an initial condition $\mathbf{x}_0$ on or near an attractor of (1). Let $\mathbf{x}_s(t)$ be the solution of (1) with $\mathbf{x}_s(0) = \mathbf{x}_0$. Let $g(f, \mathbf{x}_0, t)$ denote a stable numerical algorithm that approximates the solution to (1) (i.e. Python's `scipy.integrate.odeint()`). That is,

$$g(f, \mathbf{x}_0, t) \approx \mathbf{x}_s(t). \tag{C2}$$

Let ϵ be a small positive number and choose a vector $\mathbf{y}_0 \neq \mathbf{x}_0$. For $i = 0, 1, \ldots, n$, define

$$d_i := ||\mathbf{y}_i - \mathbf{x}_i|| \qquad \text{and} \qquad \mathbf{y}_i^* := \mathbf{x}_i + \frac{\epsilon}{d_i}\left(\mathbf{y}_i - \mathbf{x}_i\right),$$

where $\mathbf{y}_i$ is defined for $i = 1, \ldots, n$ as

$$\mathbf{y}_i := g(f, \mathbf{y}_{i-1}^*, \Delta t). \tag{C3}$$

See Figure C1 for a graphical depiction of this process.

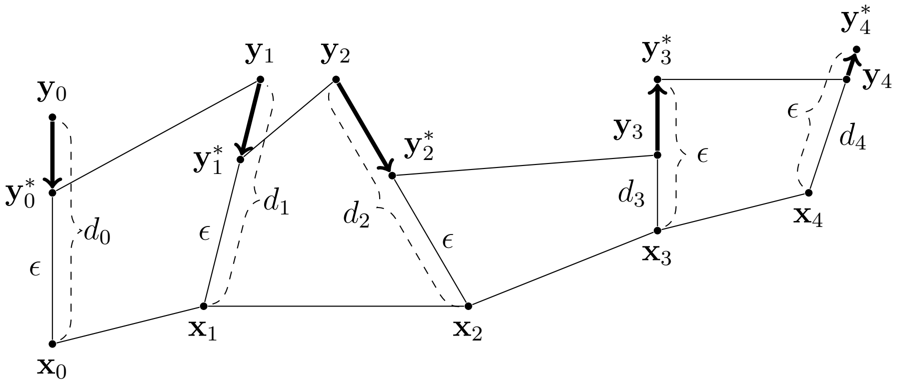

Figure C1: Cartoon of the numerical approximation of the Lyapunov exponent for the solution $\mathbf{x}_s$ of some system $\dot{\mathbf{x}} = f(\mathbf{x})$ with initial condition $\mathbf{x}(0) = \mathbf{x}_0$. The initial ϵ-perturbation of $\mathbf{x}_0$ is $\mathbf{y}_0^*$. Each $\mathbf{y}_i$ is rescaled to a distance of ϵ from $\mathbf{x}_i$.

Finally, define

$$L_i \coloneqq \ln\left(\frac{d_i}{\epsilon}\right). \tag{C4}$$

If $L_i > 0$ (< 0), then nearby solutions at that point move away from (towards) the reference solution $\mathbf{x}_s$, indicating chaotic (stable) dynamics. Then the Lyapunov exponenet L for the reference trajectory $\mathbf{x}_s$ is defined as

$$L \coloneqq \frac{1}{n}\sum_{i=1}^{n} L_i. \tag{C5}$$

If $L < 0$, we say $\dot{\mathbf{x}} = f(\mathbf{x})$ is stable around $\mathbf{x}_s$ (and that $\mathbf{x}_s$ is a stable trajectory). If $L > 0$, we say $\dot{\mathbf{x}} = f(\mathbf{x})$ is chaotic around $\mathbf{x}_s$ [Sprott, 2003].

Parameters for Figure 4 of the main text are in Table B3 (Appendix B).

References

J. C. Sprott. *Chaos and time-series analysis*, volume 69. Citeseer, 2003.

Appendix D: Determining the time-averaged solution of the ecological subsystem for constant traits $\overline{x}$ and $\overline{y}$

This appendix addresses the convergence of the ecological system (Equation 1a in the main text). In particular, for constant predator mean traits $\overline{x}$ and $\overline{y}$, we have

$$\begin{aligned}
\dot{N}_1 &= N_1\left(r_1\left(1-\frac{N_1}{K_1}\right)-a_1P\right)\\
\dot{N}_2 &= N_2\left(r_2\left(1-\frac{N_2}{K_2}\right)-a_2P\right)\\
\dot{P} &= P\left(a_1e_1N_1+a_2e_2N_2-d\right)
\end{aligned} \tag{D1}$$

where $a_i \coloneqq \overline{a}_i(\overline{x})$ and $e_i \coloneqq b_i - m_i c_i \overline{S}_i(\overline{y})$. We assume $e_i > 0$ for the remainder of this appendix.

First, we nondimensionalize (D1):

$$\begin{aligned}
\dot{u}_1 &= u_1\left(r_1-u_1-a_1y\right)\\
\dot{u}_2 &= u_2\left(r_2-u_2-a_2y\right)\\
\dot{v} &= v\left(a_1e_1u_1+a_2e_2u_2-d\right)
\end{aligned} \tag{D2}$$

In this case, there are seven ecologically relevant equilibria of (D2):

$$
\begin{aligned}
(E_{+++}) &= \frac{1}{|\Theta|}\left(\tilde{u}_1, \tilde{u}_2, \tilde{v}\right), \text{ where} \\
\tilde{u}_1 &= a_1 d + a_2 e_2 \left(a_2 r_1 - a_1 r_2\right) \\
\tilde{u}_2 &= a_2 d + a_1 e_1 \left(a_1 r_2 - a_2 r_1\right) \\
\tilde{v} &= a_1 e_1 r_1 + a_2 e_2 r_2 - d \\
|\Theta| &= a_1^2 e_1 + a_2^2 e_2 \\
(E_{+0+}) &= \left(\frac{d}{a_1 e_1}, 0, \frac{1}{a_1}\left(r_1 - \frac{d}{a_1 e_1}\right)\right) \\
(E_{0++}) &= \left(0, \frac{d}{a_2 e_2}, \frac{1}{a_2}\left(r_2 - \frac{d}{a_2 e_2}\right)\right) \\
(E_{++0}) &= (r_1, r_2, 0) \\
(E_{+00}) &= (r_1, 0, 0) \\
(E_{0+0}) &= (0, r_2, 0) \\
(E_{000}) &= (0, 0, 0)
\end{aligned}
$$

The null equilibrium (E_{000}) and the one-species equilibria (E_{+00}) and (E_{0+0}) are unstable. Because it is impossible for any two of $\tilde{u}_1$, $\tilde{u}_2$, and $\tilde{v}$ to be simultaneously negative, and because $|\Theta| > 0$, the basin of attraction for the two-species equilibria (E_{+0+}), (E_{0++}), and (E_{++0}) contains $(0, \infty)^3$ if $\tilde{u}_2 < 0$, $\tilde{u}_1 < 0$, and $\tilde{v} < 0$, respectively. If (E_{+++}) is positive, then all boundary equilibria are unstable (in particular, the per-capita growth rate of at least one missing species is positive), and thus all trajectories with positive initial conditions remain in the interior, eventually bounded away from the boundary $u_1 u_2 v = 0$. In this case, since (D2) is dissipative, the time-average of the trajectory approaches the unique coexistence equilibrium (E_{+++}) as $t \to \infty$ [Theorem 5.2.3, Hofbauer and Sigmund, 1998]. Thus in order to determine which equilibrium is the unique time-average for all trajectories with position initial conditions, it suffices to check the signs of $\tilde{u}_1$, $\tilde{u}_2$, and $\tilde{v}$.

References

J. Hofbauer and K. Sigmund. *Evolutionary games and population dynamics.* Cambridge university press, 1998.

Appendix E: Unimodality condition for $\overline{W}$ with respect to $\overline{y}$

In this appendix, we assume the widths of the two susceptibility curves are equal: $\tau := \tau_1 = \tau_2$. The predator fitness is thus

$$\overline{W} = \left(b_1 - m_1 c_1 \overline{S}_1(\overline{y})\right) \overline{a}_1(\overline{x}) N_1 + \left(b_2 - m_2 c_2 \overline{S}_2(\overline{y})\right) \overline{a}_2(\overline{x}) N_2 - d. \tag{E1}$$

where

$$\overline{S}_i(\overline{y}) = \beta_i - \frac{(\beta_i - \gamma_i)\tau}{\sqrt{\sigma_y^2 + \tau^2}} \exp\left[-\frac{(\overline{y} - \phi_i)^2}{2\left(\sigma_y^2 + \tau^2\right)}\right], \qquad i = 1, 2.$$

Thus, $\overline{y}$ fitness gradient is

$$\frac{\partial \overline{W}}{\partial \overline{y}} = -m_1 c_1 \overline{S}_1'(\overline{y}) \overline{a}_1(\overline{x}) N_1 - m_2 c_2 \overline{S}_2'(\overline{y}) \overline{a}_2(\overline{x}) N_2 \tag{E2}$$

$$= m_1 c_1 \left(\frac{(\beta_1 - \gamma_1)\tau(\phi_1 - \overline{y})}{(\sigma_y^2 + \tau^2)^{\frac{3}{2}}} \exp\left[-\frac{(\overline{y} - \phi_1)^2}{2\left(\sigma_y^2 + \tau^2\right)}\right]\right) \overline{a}_1(\overline{x}) N_1 + m_2 c_2 \left(\frac{(\beta_2 - \gamma_2)\tau(\phi_2 - \overline{y})}{(\sigma_y^2 + \tau^2)^{\frac{3}{2}}} \exp\left[-\frac{(\overline{y} - \phi_2)^2}{2\left(\sigma_y^2 + \tau^2\right)}\right]\right) \overline{a}_2(\overline{x}) N_2 \tag{E3}$$

For simplicity, we introduce a composite parameter $A := \sigma_y^2 + \tau^2$ and $Z_i := \dfrac{m_i c_i (\beta_i - \gamma_i)\tau \overline{a}_i(\overline{x}) N_i}{A^{\frac{3}{2}}}$ and so

$$\frac{\partial \overline{W}}{\partial \overline{y}} = Z_1(\phi_1 - \overline{y}) \exp\left[-\frac{(\overline{y} - \phi_1)^2}{2A}\right] + Z_2(\phi_2 - \overline{y}) \exp\left[-\frac{(\overline{y} - \phi_2)^2}{2A}\right] \tag{E4}$$

We also rescale so that $\phi_1 = 0$, $\phi_2 = \phi$, and $\overline{y} = \tilde{y}\phi$. Thus,

$$\frac{\partial \overline{W}}{\partial \overline{y}} = -Z_1 \phi \tilde{y} \exp\left[-\frac{\tilde{y}^2\phi^2}{2A}\right] + Z_2\phi(1-\tilde{y})\exp\left[-\frac{\phi^2(\tilde{y}-1)^2}{2A}\right] \tag{E5}$$

To find critical points, we set $\frac{\mathrm{d}\overline{W}}{\mathrm{d}\overline{y}} = 0$:

$$Z_1\tilde{y}\exp\left[-\frac{\tilde{y}^2\phi^2}{2A}\right] = Z_2(1-\tilde{y})\exp\left[-\frac{\phi^2(\tilde{y}-1)^2}{2A}\right] \tag{E6}$$

Let $\Psi := \dfrac{Z_2}{Z_1} = \dfrac{m_2 c_2(\beta_2-\gamma_2)\overline{a}_2(\overline{x})N_2}{m_1 c_1(\beta_1-\gamma_1)\overline{a}_1(\overline{x})N_1}$, the ratio of the differences between maximally and minimally effective individual parasites. With this notation, we have

$$\exp\left[-\frac{\phi^2\left(\tilde{y}-\frac{1}{2}\right)}{A}\right] = \Psi\left(\frac{1}{\tilde{y}}-1\right) \tag{E7}$$

This is the same form as Equation (A2) from Appendix A in Patel and Schreiber [2015]. The remainder of this appendix is a restatement of their analytical results applied to this model.

Thus, if $\Psi = 1$, then $\tilde{y} = \frac{1}{2}$ is always a critical point. If $\phi^2 < 4A$, this point is stable and if $\phi^2 > 4A$, this point is unstable. Graphical analysis shows that two additional stable equilibria exist if $\phi^2 > 4A$ (one less than $\frac{1}{2}$ and one more than $\frac{1}{2}$), and so the fitness function is bimodal when $\phi^2 > 4A$, and this corresponds to a pitchfork bifurcation.

If $\Psi \neq 1$, then the symmetry of the bifurcation breaks. If $\Psi < 1$, then parasite 1 has a greater effect on predator fitness than the parasite 2, and so we predict the critical points to be closer to $\tilde{y} = 0$ so the predator is less susceptible to limnetic parasitism. If $\Psi > 1$, we likewise expect the critical points to be closer to $\tilde{y} = 1$. We can check this by solving for $\frac{\mathrm{d}\tilde{y}}{\mathrm{d}\Psi}$:

$$\frac{\mathrm{d}\tilde{y}}{\mathrm{d}\Psi} = \frac{\dfrac{1}{\tilde{y}}-1}{\dfrac{\Psi}{\tilde{y}^2} - \dfrac{\phi^2}{A}\exp\left[-\dfrac{\phi^2\left(\tilde{y}-\frac{1}{2}\right)}{A}\right]}. \tag{E8}$$

For all relevant values of $\tilde{y}$ ($\tilde{y} \in (0,1)$), the numerator is positive. The denominator is

positive for stable critical points and thus $\frac{d\tilde{y}}{d\Psi} > 0$ for stable critical points in $(0, 1)$. Thus, the stable phenotype values of $\tilde{y}$ increase as Ψ increases.

References

S. Patel and S. J. Schreiber. Evolutionarily driven shifts in communities with intraguild predation. *The American Naturalist*, 186(5):E98–E110, nov 2015. doi: 10.1086/683170. URL `https://doi.org/10.1086/683170`.

Sick of Eating: eco-evo-immuno dynamics of predators and their trophically acquired parasites

Appendix F: Model with Epidemiological Component

Let N_{iS} be the density of susceptible (uninfected) prey i and let N_{iI} be the density of infected prey i. Let L_i be the parasite load generated by the predator after consuming infected prey i. Let μ_i denote the rate of background parasite migration from all sources. Let ℓ_i be the rate at which the predator converts consumed parasite i into new parasites. So $L_i = \mu_i + \ell_i \bar{a}_i(\bar{x})\bar{S}_i(\bar{y})PN_{iI}$, the total predator population infected with parasite i after consuming infected prey i. Susceptible prey i become infected at a rate ν_i proportional to L_i. The parasite is not passed vertically, and thus the prey i dynamics are:

$$\frac{\mathrm{d}N_{iS}}{\mathrm{d}t} = r_i N_i - \frac{r_i}{K_i} N_i N_{iS} - \bar{a}_i(\bar{x})PN_{iS} - \nu_i L_i N_{iS}$$
$$\frac{\mathrm{d}N_{iI}}{\mathrm{d}t} = \nu_i L_i N_{iS} - \frac{r_i}{K_i} N_i N_{iI} - \bar{a}_i(\bar{x})PN_{iI}$$

Define $N_i := N_{iS} + N_{iI}$ as the total prey i density. The proportion of prey i which are

infected, c_i, is now longer constant, but a dynamical variable: $c_i := \frac{N_{iI}}{N_i}$. Thus,

$$\frac{\mathrm{d}N_i}{\mathrm{d}t} = N_i\left(r_i\left(1 - \frac{N_i}{K_i}\right) - \bar{a}_i(\bar{x})P\right), \quad i = 1, 2 \tag{F1}$$

$$\begin{aligned}
\frac{\mathrm{d}c_i}{\mathrm{d}t} &= \frac{\mathrm{d}}{\mathrm{d}t}\left(\frac{N_{iI}}{N_i}\right) \\
&= \frac{1}{N_i^2}\Bigg[N_i\left(\nu_i L_i N_{iS} - \frac{r_i}{K_i}N_i N_{iI} - \bar{a}_i(\bar{x})PN_{iI}\right) \\
&\qquad\qquad - N_{iI}N_i\left(r_i\left(1 - \frac{N_i}{K_i}\right) - \bar{a}_i(\bar{x})P\right)\Bigg] \\
&= \frac{1}{N_i}[\nu_i L_i N_{iS} - r_i N_{iI}] \\
&= \nu_i L_i(1 - c_i) - r_i c_i \\
&= \nu_i\big(\mu_i + \ell_i \bar{a}_i(\bar{x})\bar{S}_i(\bar{y})PN_{iI}\big)(1 - c_i) - r_i c_i \\
&= \nu_i\big(\mu_i + \ell_i \bar{a}_i(\bar{x})\bar{S}_i(\bar{y})PN_i c_i\big)(1 - c_i) - r_i c_i, \quad i = 1, 2
\end{aligned} \tag{F2}$$

When $\ell_i = 0$, $c_i \to c_i^* = \dfrac{\nu_i \mu_i}{r_i + \nu_i \mu_i}$ as $t \to \infty$. If $c_i = c_i^*$, then this model is identical to model (1a,b) from the main text since $\frac{\mathrm{d}c_i}{\mathrm{d}t}\Big|_{c_i = c_i^*} = 0$. The predator density and trait equations remain unchanged, of course noting that c_i is a dynamical variable rather than a constant:

$$\frac{\mathrm{d}P}{\mathrm{d}t} = P\left(\sum_{i=1}^{2}\left[\big(b_i - m_i c_i \bar{S}_i(\bar{y})\big)\bar{a}_i(\bar{x})N_i\right] - d\right) \tag{F3}$$

$$\frac{\mathrm{d}\bar{x}}{\mathrm{d}t} = \sigma_{x,G}^2 \frac{\partial\left(\frac{1}{P}\frac{\mathrm{d}P}{\mathrm{d}t}\right)}{\partial \bar{x}} \tag{F4}$$

$$\frac{\mathrm{d}\bar{y}}{\mathrm{d}t} = \sigma_{y,G}^2 \frac{\partial\left(\frac{1}{P}\frac{\mathrm{d}P}{\mathrm{d}t}\right)}{\partial \bar{y}} \tag{F5}$$

We ran 4,000 simulations of Model (F1-5) using Latin hypercube sampling. Parameters were chosen identically to those chosen for model (2) in the main text. In particular, parameters are given in Table B3 in Appendix B, except c_i are dynamical variables and are thus determined by the Model rather than being chosen at random.

As ℓ increases, a few patterns emerge that are different than what is seen when $\ell = 0$ (Figures 3 and 4). First, in cases in which the immune tradeoff is strong, intermediate

immune traits are much more likely to evolve than when $\ell = 0$ (Figure 3). Second, the relationship between prey intake and parasite exposure seems to approach a linear relationship with drastic reductions in variability, regardless of strength of foraging and immune tradeoffs. Unlike when $\ell = 0$ (as in the main text), our results suggest that any nonlinear relationship between prey intake and parasite infection is caused by immune evolution and not by fluctuations in parasite abundance due to local recruitment, regardless of the strength of foraging and immune tradeoffs. This contrasts when the results summarized in Figure 4, which show that evolution of immunity causes nonlinear relationships between prey intake and parasite infection only when immune tradeoffs are weak. The following six figures recreate Figure 3 and 4, but $\ell = 1$ (Figures F1 and F2), $\ell = 10$ (Figures F3 and F4), and $\ell = 100$ (Figures F5 and F6).

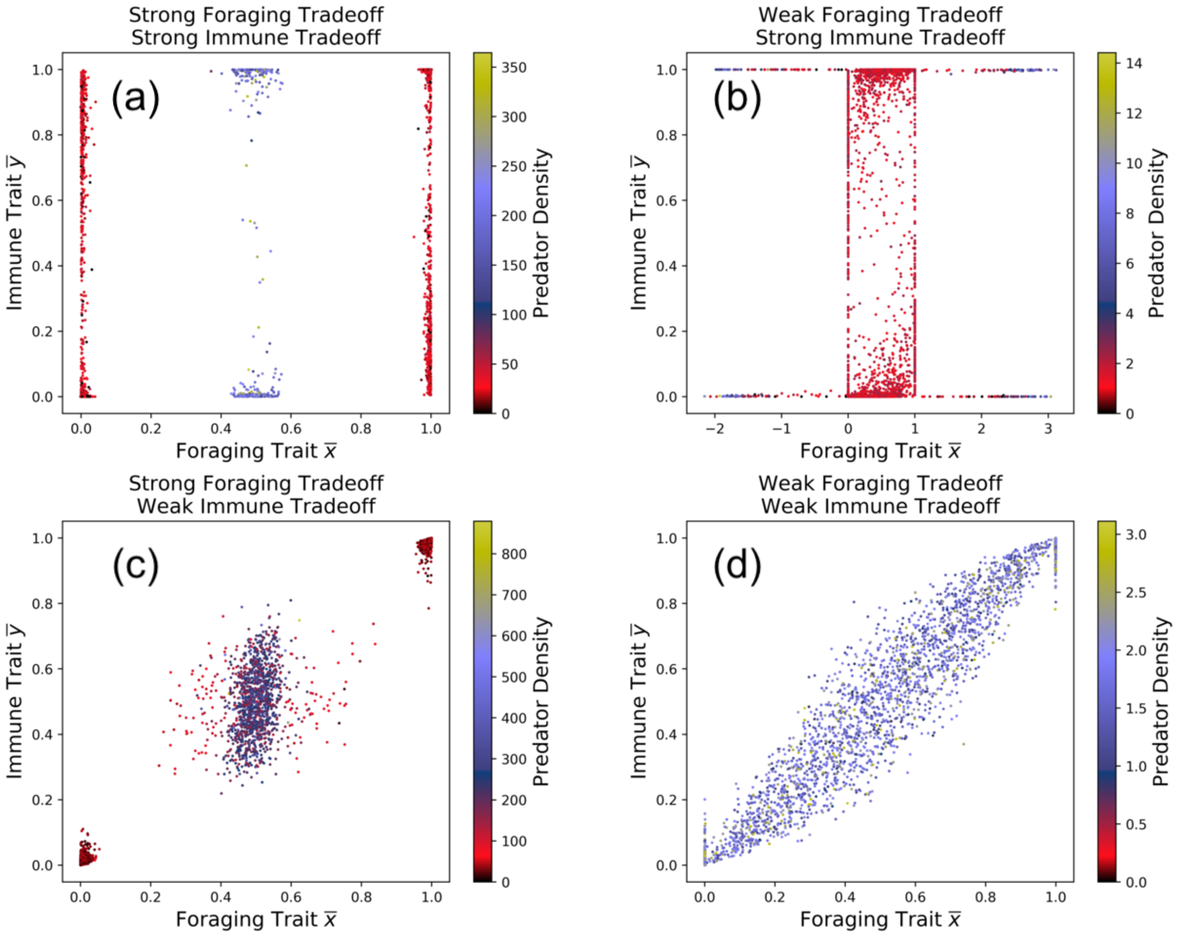

Figure F1: Locations of Stable equilibria for a Latin Hypercube sample of parameter space for $\ell_1 = \ell_2 = 1$. In (a) and (b) immune tradeoffs are strong ($\tau_i = 0.01$) and in (c) and (d) immune tradeoffs are weak ($\tau_i = 1$). In (a) and (c), foraging tradeoffs are strong ($\zeta_i = 0.01$) and in (b) and (d) foraging tradeoffs are weak ($\zeta_i = 1$). The color of each dot represents the density of the predator population at the evolutionary equilibrium.

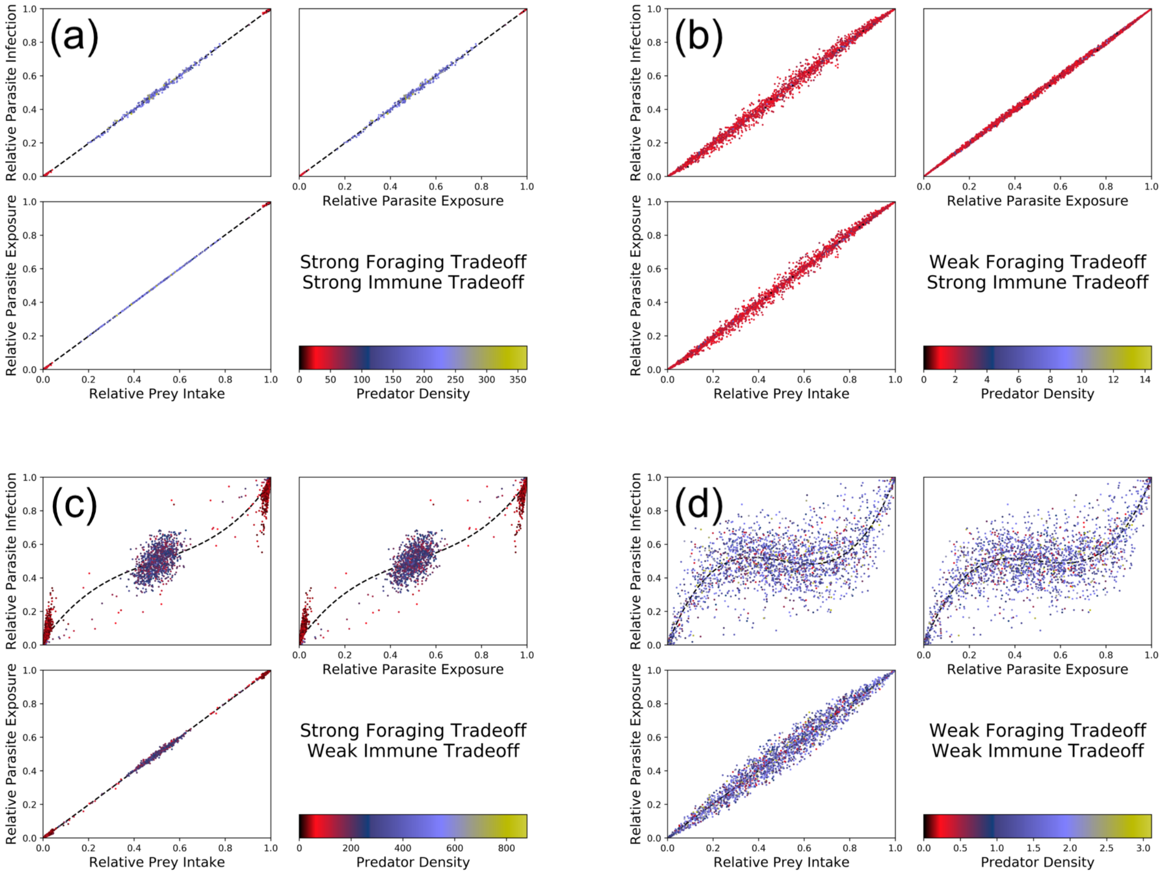

Figure F2: Prey intake, parasite exposure, and parasite infection over the same subset of parameter space given in Figure F1. The dots are colored as in Figure F1. The dashed lines are splines of the data, included in order to better identify patterns between prey intake, parasite exposure, and parasite infection. In (a) and (b) immune tradeoffs are strong ($\tau_i = 0.01$) and in (c) and (d) immune tradeoffs are weak ($\tau_i = 1$). In (a) and (c), foraging tradeoffs are strong ($\zeta_i = 0.01$) and in (b) and (d) foraging tradeoffs are weak ($\zeta_i = 1$).

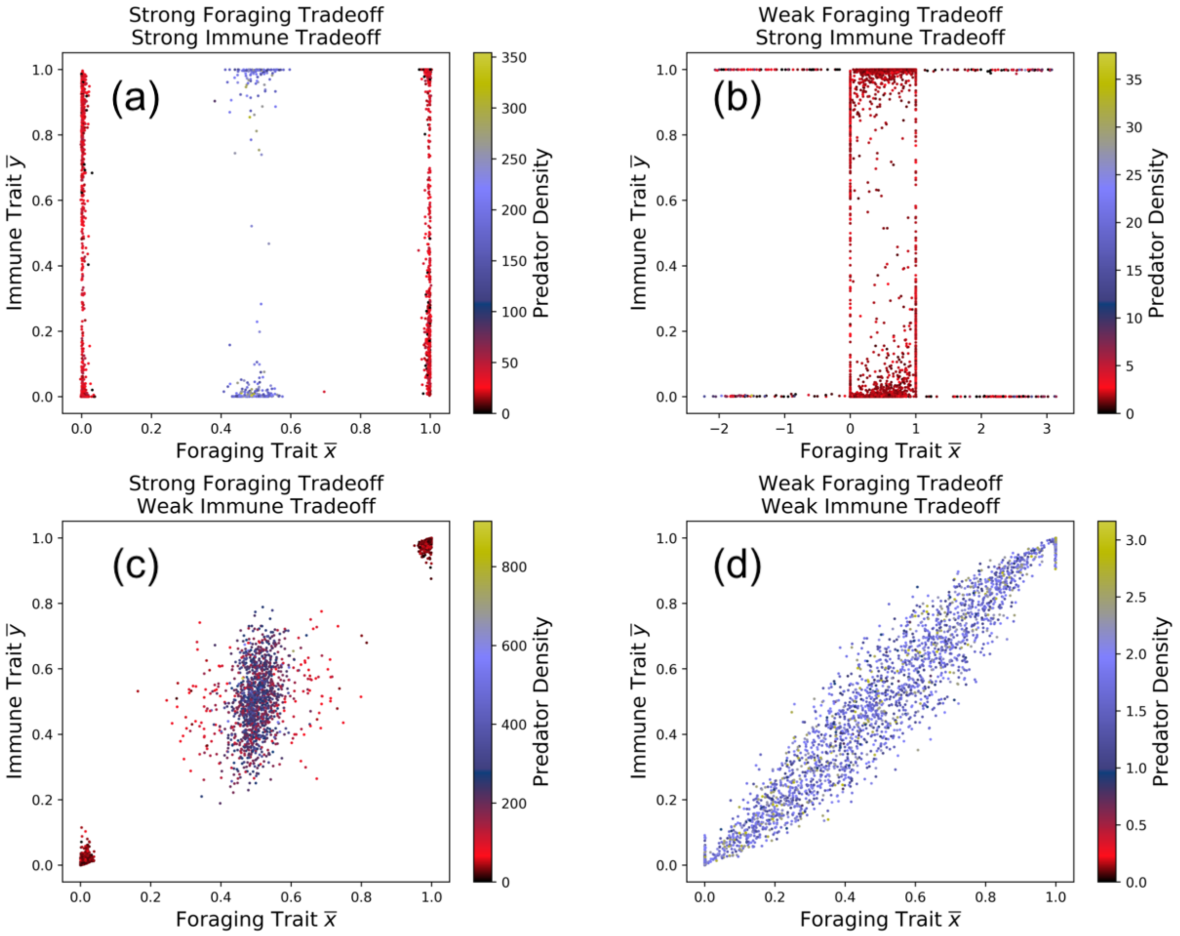

Figure F3: Locations of Stable equilibria for a Latin Hypercube sample of parameter space for $\ell_1 = \ell_2 = 10$. In (a) and (b) immune tradeoffs are strong ($\tau_i = 0.01$) and in (c) and (d) immune tradeoffs are weak ($\tau_i = 1$). In (a) and (c), foraging tradeoffs are strong ($\zeta_i = 0.01$) and in (b) and (d) foraging tradeoffs are weak ($\zeta_i = 1$). The color of each dot represents the density of the predator population at the evolutionary equilibrium.

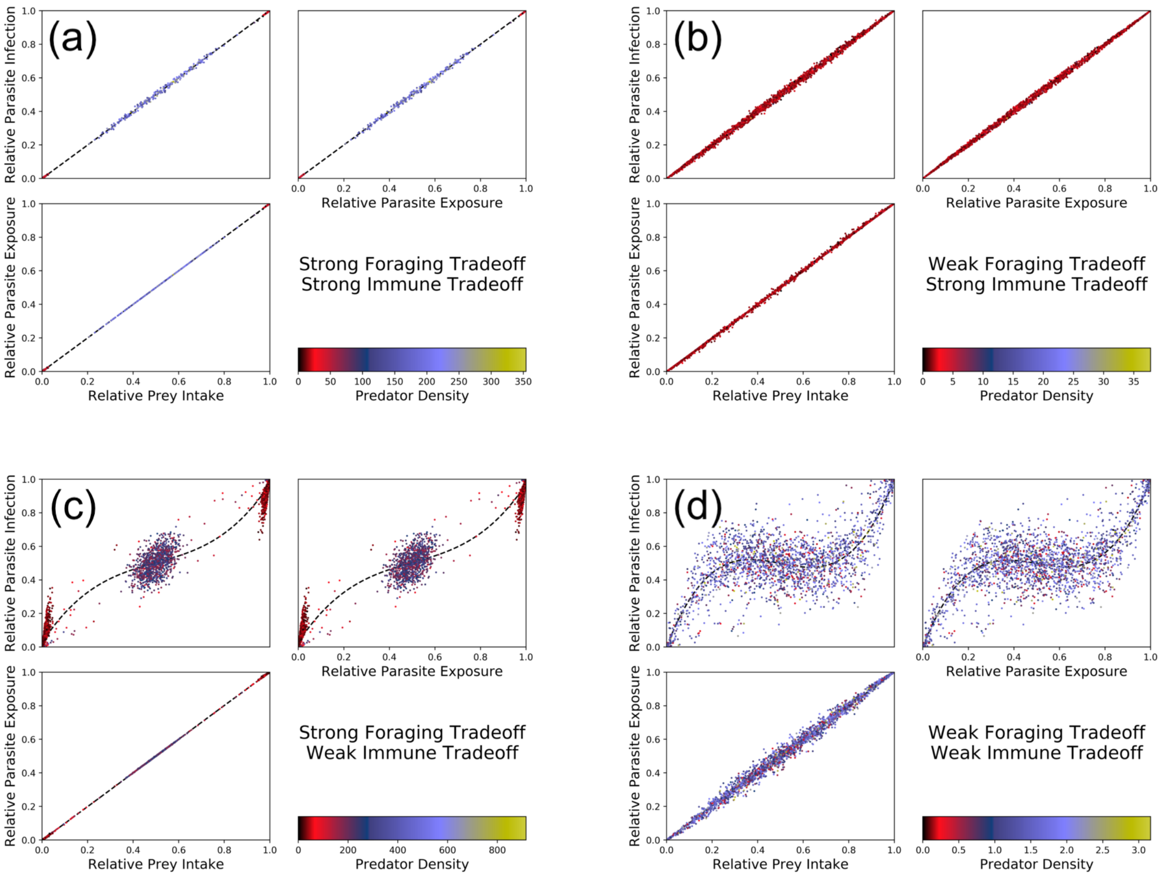

Figure F4: Prey intake, parasite exposure, and parasite infection over the same subset of parameter space given in Figure F3. The dots are colored as in Figure F3. The dashed lines are splines of the data, included in order to better identify patterns between prey intake, parasite exposure, and parasite infection. In (a) and (b) immune tradeoffs are strong ($\tau_i = 0.01$) and in (c) and (d) immune tradeoffs are weak ($\tau_i = 1$). In (a) and (c), foraging tradeoffs are strong ($\zeta_i = 0.01$) and in (b) and (d) foraging tradeoffs are weak ($\zeta_i = 1$).

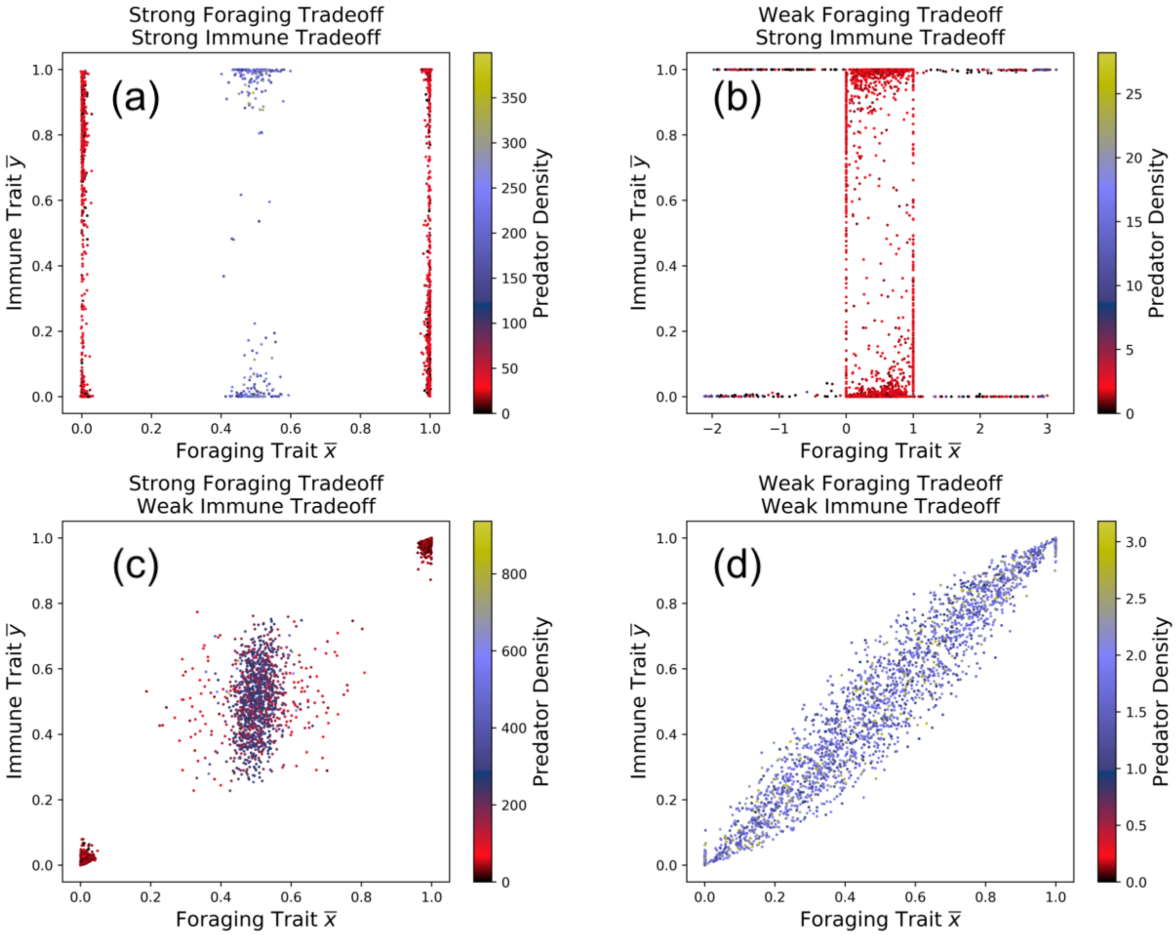

Figure F5: Locations of Stable equilibria for a Latin Hypercube sample of parameter space for $\ell_1 = \ell_2 = 100$. In (a) and (b) immune tradeoffs are strong ($\tau_i = 0.01$) and in (c) and (d) immune tradeoffs are weak ($\tau_i = 1$). In (a) and (c), foraging tradeoffs are strong ($\zeta_i = 0.01$) and in (b) and (d) foraging tradeoffs are weak ($\zeta_i = 1$). The color of each dot represents the density of the predator population at the evolutionary equilibrium.

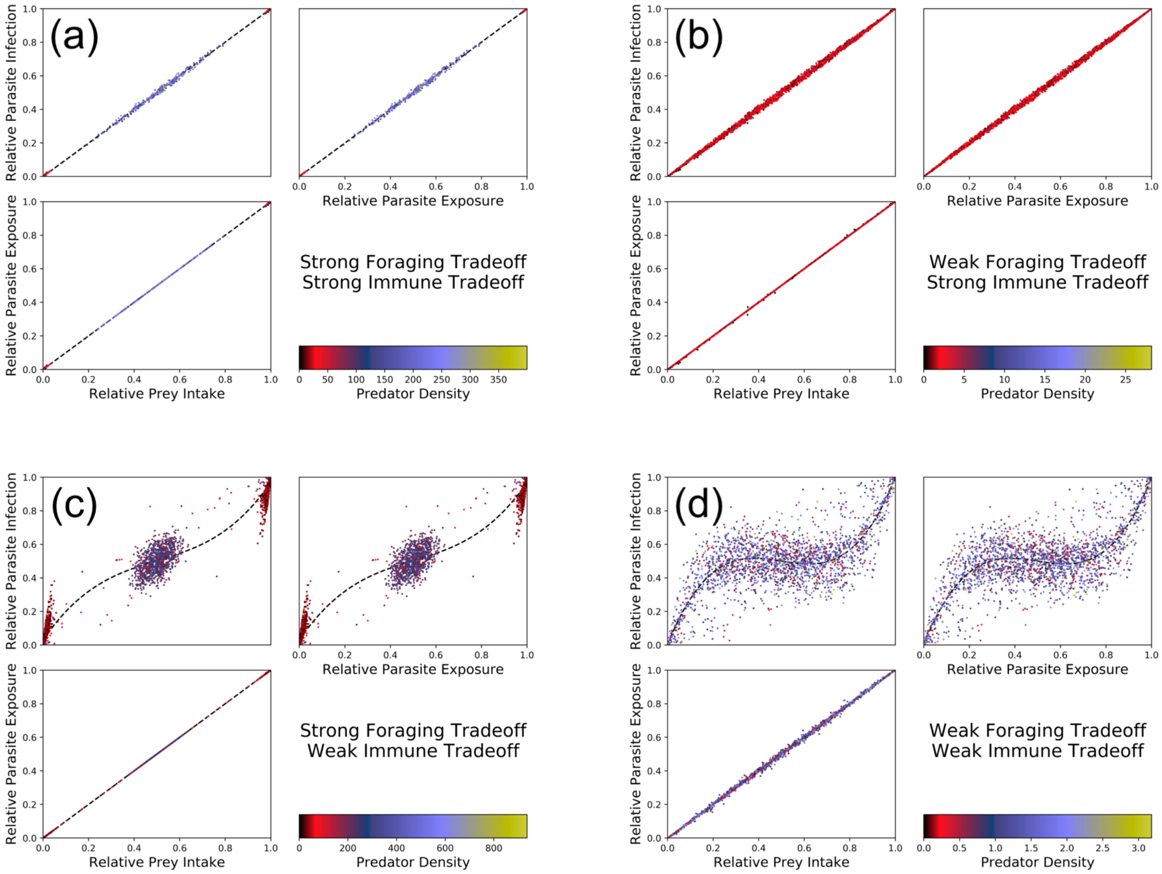

Figure F6: Prey intake, parasite exposure, and parasite infection over the same subset of parameter space given in Figure F5. The dots are colored as in Figure F5. The dashed lines are splines of the data, included in order to better identify patterns between prey intake, parasite exposure, and parasite infection. In (a) and (b) immune tradeoffs are strong ($\tau_i = 0.01$) and in (c) and (d) immune tradeoffs are weak ($\tau_i = 1$). In (a) and (c), foraging tradeoffs are strong ($\zeta_i = 0.01$) and in (b) and (d) foraging tradeoffs are weak ($\zeta_i = 1$).

Sick of Eating: eco-evo-immuno dynamics of predators and their trophically acquired parasites

Appendix G: Prey intake, parasite exposure, and parasite infection, conditional on coexistence

Table 1 shows the number of simulations resulting in non-coexistence. In the weak foraging tradeoff regime, one or the other prey may be excluded. In the strong foraging tradeoff regime, the predator may be excluded. In the weak foraging tradeoff regime, although a large percentage of the simulations result in non-coexistence, the relationships described in the main text between prey intake, parasite exposure, and parasite infection remain unchanged. This is because relative prey intake, parasite exposure, and parasite infection each approach 0 or 1 as $t \to \infty$ when one or other prey is excluded. In the strong foraging tradeoff regime, only a small percentage of simulations result in non-coexistence, and the relationships remain the same when those cases are excluded from the analysis. Figure G1 shows these relationships, conditional on all three species coexisting. Figure G2 shows the conditions under which one or the other prey is excluded in the weak foraging tradeoff regime.

	N_1 excluded	N_2 excluded	P excluded	Total
Weak foraging, weak immune	702/4000	739/4000	0/4000	1441/4000
Weak foraging, strong immune	688/4000	727/4000	0/4000	1415/4000
Strong foraging, weak immune	0/4000	0/4000	15/4000	15/4000
Strong foraging, strong immune	0/4000	0/4000	66/4000	66/4000

Table 1: The numbers of simulations which result in non-coexistence in each of the four scenarios (weak and strong foraging and immune tradeoffs). There are no cases in which more than one population is excluded.

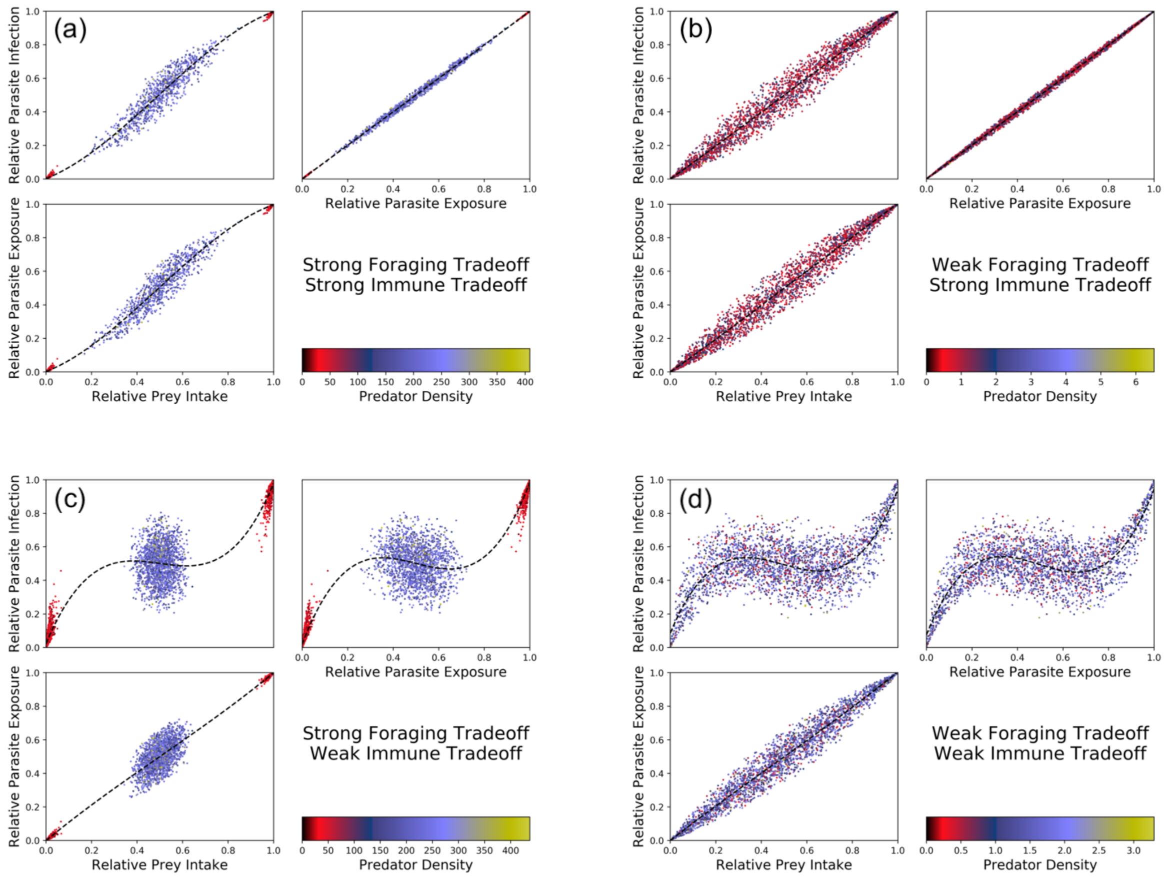

Figure G1: Prey intake, parasite exposure, and parasite infection over a subset of parameter space, *conditional on all three species coexisting.* The dots are colored as in the main text and in Appendix F. The dashed lines are splines of the data, included in order to better identify patterns between prey intake, parasite exposure, and parasite infection. In (a) and (b) immune tradeoffs are strong ($\tau_i = 0.01$) and in (c) and (d) immune tradeoffs are weak ($\tau_i = 1$). In (a) and (c), foraging tradeoffs are strong ($\zeta_i = 0.01$) and in (b) and (d) foraging tradeoffs are weak ($\zeta_i = 1$). The color of each dot represents the density of the predator population at the evolutionary equilibrium.

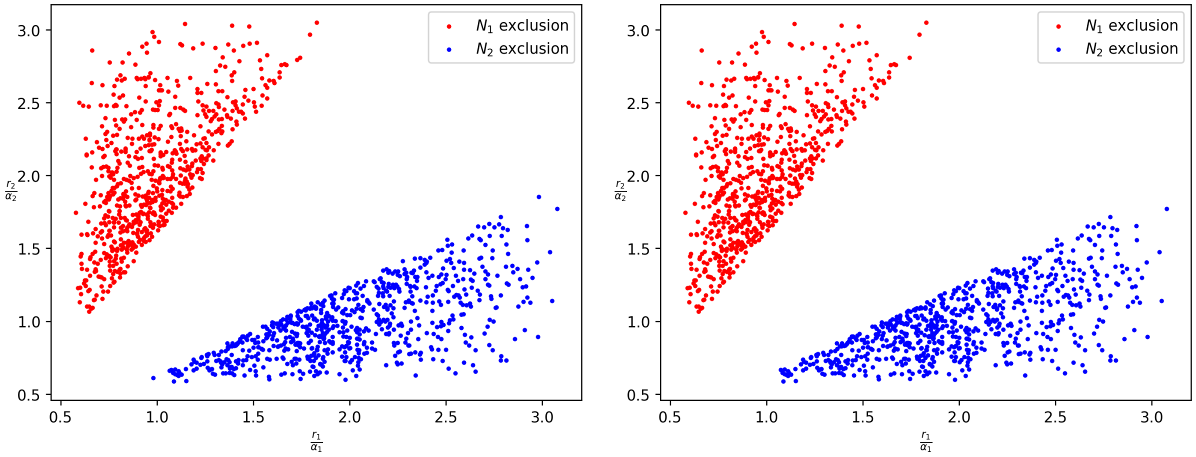

Figure G2: The equilibrium conditions of $\frac{r_1}{\bar{a}_1}$ and $\frac{r_2}{\bar{a}_2}$ over a subset of parameter space, *conditional on one species being excluded*. When $\frac{r_1}{\bar{a}_1}$ is small (large) in comparison to $\frac{r_2}{\bar{a}_2}$, N_1 (N_2) is excluded, and the predator consumes only N_2 (N_1). On the left, both foraging and immune tradeoffs are weak. On the right, the foraging tradeoff is weak and the immune tradeoff is strong. In the weak foraging tradeoff regime, the predator is never excluded.

Sick of Eating: eco-evo-immuno dynamics of predators and their trophically acquired parasites

Appendix H: Coexistence Conditions

In this appendix, we derive conditions to ensure all three species coexist, in the sense of permanence [Hutson and Schmitt, 1992, Schreiber, 2000, Patel and Schreiber, 2018], for the eco-evo-immuno model

$$\begin{aligned}
\frac{\mathrm{d}P}{\mathrm{d}t} &= P\overline{W}(\overline{x}, \overline{y}, P, N_1, N_2), \\
\frac{\mathrm{d}N_i}{\mathrm{d}t} &= N_i\overline{Y}_i(\overline{x}, P, N_1, N_2), \qquad i = 1, 2. \\
\frac{\mathrm{d}\overline{x}}{\mathrm{d}t} &= \sigma^2_{x,G}\frac{\partial \overline{W}}{\partial \overline{x}} \\
\frac{\mathrm{d}\overline{y}}{\mathrm{d}t} &= \sigma^2_{y,G}\frac{\partial \overline{W}}{\partial \overline{y}}
\end{aligned} \tag{H1}$$

presented in the main text. Define I_θ and I_ψ to be the closed intervals determined by θ_1, θ_2 and ψ_1, ψ_2, respectively. For example, if $\theta_2 > \theta_1$, then $I_\theta = [\theta_1, \theta_2]$. As the region $[0, \infty)^3 \times I_\theta \times I_\psi$ is forward invariant for the solutions of (H1), we only consider initial conditions in this forward invariant region. We say that (H1) is permanent if there exists $M > m > 0$ such that for all initial conditions $(N_1(0), N_2(0), P(0), x(0), y(0)) \in (0, \infty)^3 \times I_\theta \times I_\psi$

$$M \geq \limsup_{t\to\infty} \max\{N_1(t), N_2(t), P(t)\} \geq \liminf_{t\to\infty} \min\{N_1(t), N_2(t), P(t)\} \geq m.$$

We use an approach for verifying permanence developed by [Schreiber, 2000] for Kolmogorov differential equations and extended by [Patel and Schreiber, 2018] to models with auxiliary variables i.e. the trait variables x and y in (H1).

We focus on the case when the predator can persist on either of the prey in isolation which occurs if $(b_i - c_i m_i S_i(\psi_i))a_i(\theta_i)K_i > d$ for $i = 1, 2$ i.e. the predator has a positive low density per-capita growth rate when it is adapted to prey i and the prey is at its carrying capacity. Under this standing assumption, the subsystem consisting of the predator and prey species i has a globally stable positive equilibrium given by $(N_i, P) = (N_i^*, P_i^*)$ where

$$\begin{aligned} N_i^* =& \frac{d}{(b_i - c_i m_i S_i(\psi_i))a_i(\theta_i)} \\ P_i^* =& \frac{r_i}{a_i(\theta_i)}(1 - N_i^*/K_i). \end{aligned} \tag{H2}$$

A proof of this assertion follows from the global stability of this equilibrium in the two dimensional region $\{(N_1, N_2, P, x, y) : N_i = 0, N_j \geq 0, P \geq 0, x = \theta_i, y = \theta_i\}$ and the theory of asymptotically autonomous systems [Benaïm and Hirsch, 1996, Mischaikow et al., 1995].

Define

$$E = \{(\theta, \psi) \in I_\theta \times I_\psi : \sum_i (b_i - c_i m_i S_i(\psi))a_i(\theta) = d\}$$

to be the set of (θ, ψ) values such that $(K_1, K_2, 0, \psi, \theta)$ is an equilibrium of the system (H1). E is a compact, invariant set for the dynamics of (H1). The sets $(0, 0, 0) \times I_\theta \times I_\psi$ (the extinction set), $(K_1, 0, 0, \theta_1, \theta_2)$ (only prey 1), $(0, K_2, 0, \theta_2, \psi_2)$ (only prey 2), $(N_1^*, 0, P_1^*, \theta_1, \psi_1)$ (only prey 1 and predator), $(0, N_2^*, P_2^*, \theta_2, \psi_2)$ (only prey 2 and predator), and $(K_1, K_2, 0) \times E$ (only both prey) define a Morse decomposition (see [Schreiber, 2000, Patel and Schreiber, 2018] for definition) for the dynamics of (H1) restricted to the set where $N_1 N_2 P = 0$ (i.e. the extinction of at least one species).

Theorem 1 of [Patel and Schreiber, 2018] implies coexistence in the sense of robust permanence occurs if and only if each of the equilibria in this Morse decomposition has at least one species with a positive per-capita growth rate. As $r_i > 0$ for $i = 1, 2$, both prey

species have positive growth rates at the equilibria in $(0,0,0) \times I_\theta \times I_\psi$. We have assumed that the per-capita growth rate of the predator is positive at the equilibria $(K_1, 0, 0, \theta_1, \psi_1)$ and $(K_2, 0, 0, \theta_2, \psi_2)$. We also need that the predator has a positive per-capita growth rate at the equilibria in $(K_1, K_2, 0, 0) \times E$. Finding an analytical characterization of this condition is, in general, non-trivial as there can be multiple trait equilibria when $N_1 = K_1, N_2 = K_2$. Moreover, even when there is only a single trait equilibrium, it is given by the solution of a transcendental equation. That being said, our numerical explorations suggest this condition is satisfied for approximately 99.5% of the parameter space that we explored.

The final condition required for coexistence is mutual invasibility of the prey. Namely, prey i has a positive per-capita growth rate at the prey j – predator subsystem, where $i \neq j$. Equivalently,

$$r_i(1 - N_j^*/K_i) > a_i(\theta_j)P_j^* \text{ for } i \neq j \tag{H3}$$

as claimed in the main text.

References

M. Benaïm and M. W. Hirsch. Asymptotic pseudotrajectories and chain recurrent flows, with applications. *J. Dynam. Differential Equations*, 8:141–176, 1996.

V. Hutson and K. Schmitt. Permanence and the dynamics of biological systems. *Mathematical Biosciences*, 111:1–71, 1992.

K. Mischaikow, H. Smith, and H. R. Thieme. Asymptotically autonomous semiflows: Chain recurrence and Lyapunov functions. *Trans. Amer. Math. Soc.*, 347:1669–1685, 1995.

S. Patel and S. J. Schreiber. Robust permanence for ecological equations with internal and external feedbacks. *Journal of Mathematical Biology*, 77(1):79–105, 2018. doi: 10.1007/s00285-017-1187-5. URL `https://doi.org/10.1007/s00285-017-1187-5`.

S. J. Schreiber. Criteria for C^r robust permanence. *Journal of Differential Equations*, 162: 400–426, 2000.
