# Supplementary figures and images for: Sick of eating: Eco‐evo‐immuno dynamics of predators and their trophically acquired parasites
Source: Evolution. 2021 Oct 12;75(11):2842–56. doi: 10.1111/evo.14353 (PMC8985590; doi:10.1111/evo.14353)

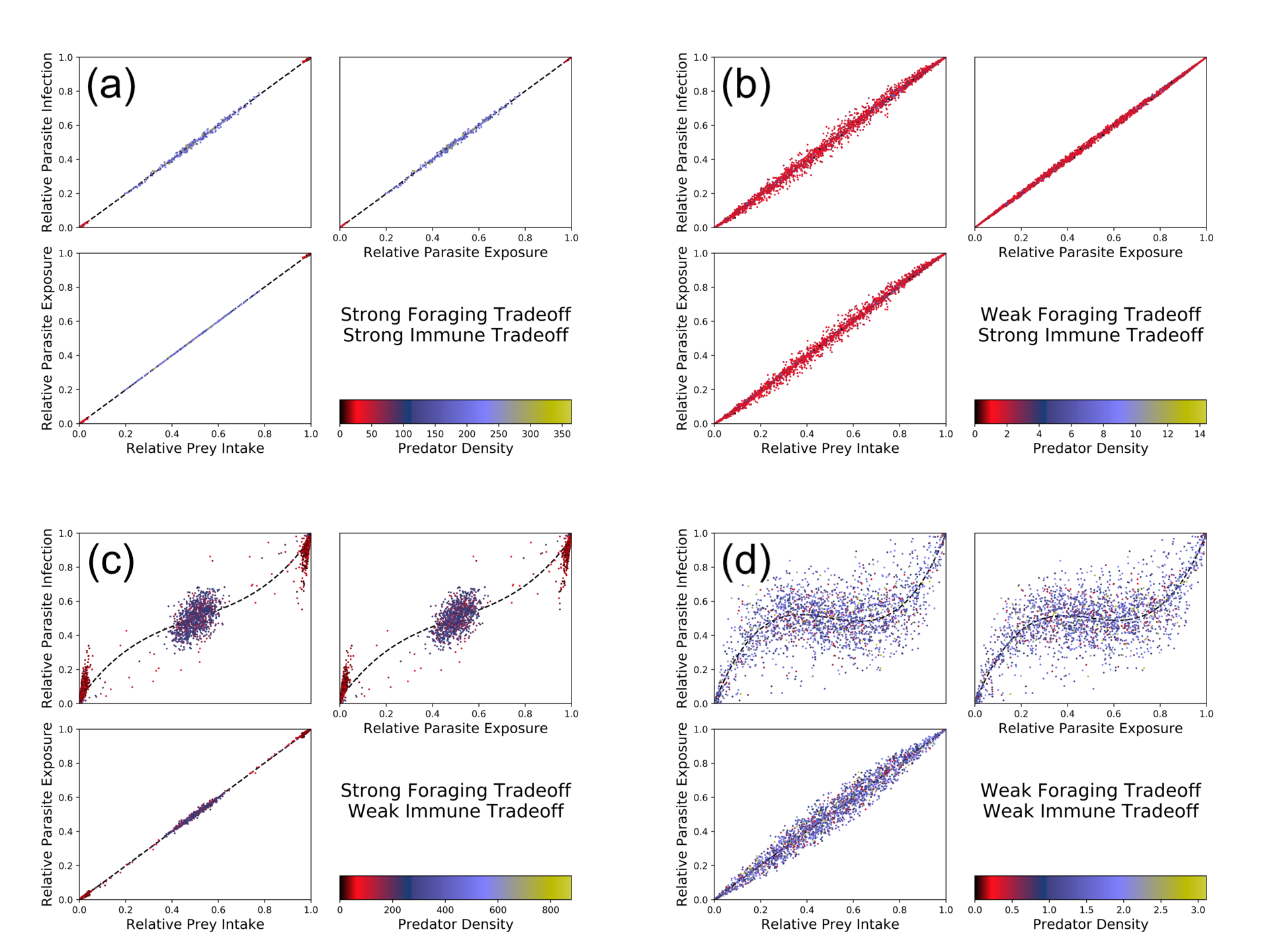

Supplement: Supplementary file 1 — Table B1: Baseline parameter values. Table B2: Figure 2 parameters. All parameters not given here are given in Table B1. Table B3: Figures 3 and 4 parameters. All parameters not given here are given in Table B1. Figure F1: Locations of Stable equilibria for a Latin Hypercube sample of parameter space for l1=l2=1. Figure F2: Prey intake, parasite exposure, and parasite infection over the same subset of parameter space given in Figure F1. Figure F3: Locations of Stable equilibria for a Latin Hypercube sample of parameter space for l1=l2=10. Figure F4: Prey intake, parasite exposure, and parasite infection over the same subset of parameter space given in Figure F3. Figure F5: Locations of Stable equilibria for a Latin Hypercube sample of parameter space for l1=l2=100. Figure F6: Prey intake, parasite exposure, and parasite infection over the same subset of parameter space given in Figure F5. Table 1: The numbers of simulations which result in noncoexistence in each of the four scenarios (weak and strong foraging and immune trade‐offs). Figure G1: Prey intake, parasite exposure, and parasite infection over a subset of parameter space, conditional on all three species coexisting. Figure G2: The equilibrium conditions of r1a¯1 and r2a¯2 over a subset of parameter space, conditional on one species being excluded. [file EVO-75-2842-s001.zip › evo14353-sup-0010-SuppMat.png]

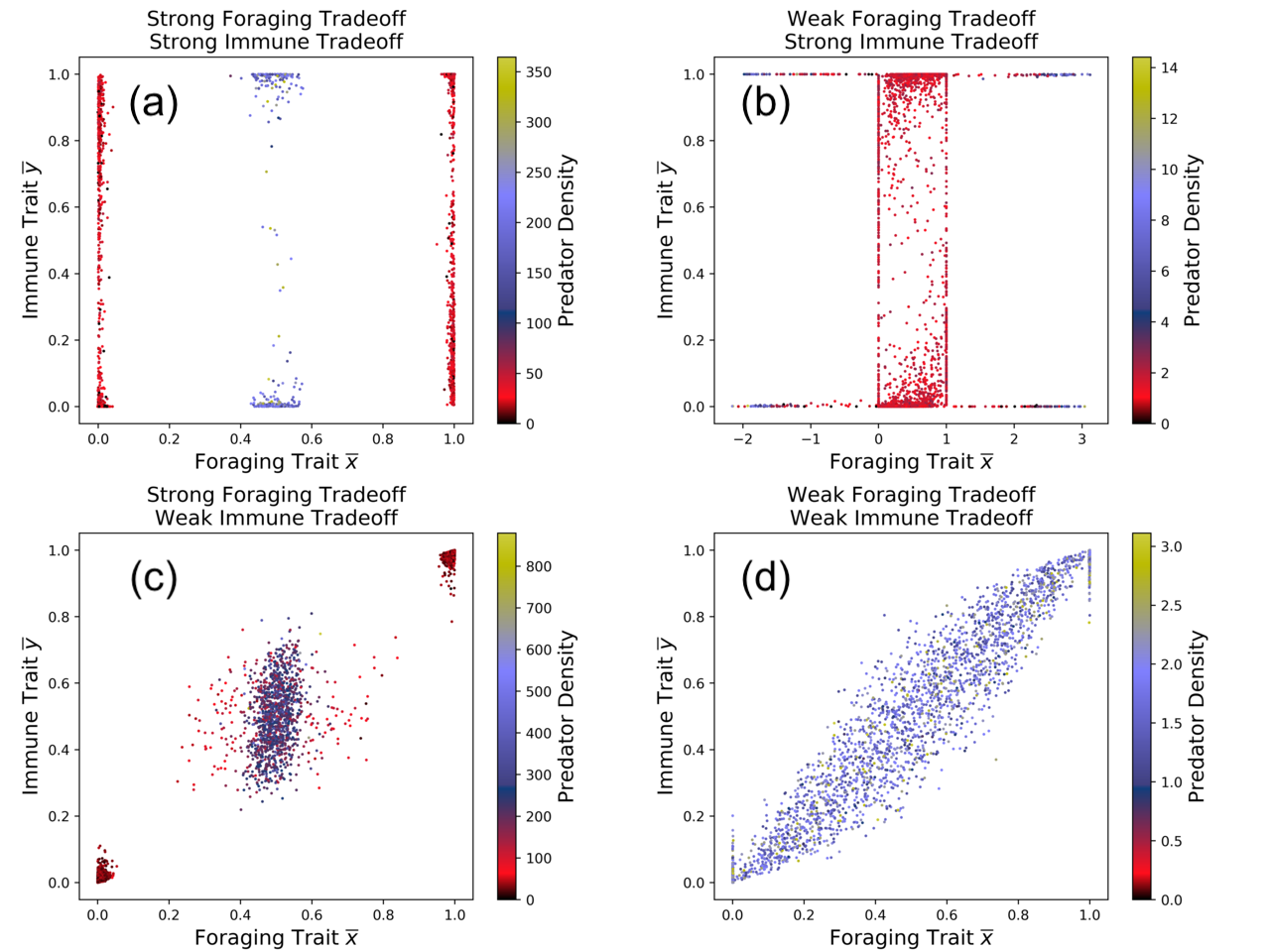

Supplement: Supplementary file 1 — Table B1: Baseline parameter values. Table B2: Figure 2 parameters. All parameters not given here are given in Table B1. Table B3: Figures 3 and 4 parameters. All parameters not given here are given in Table B1. Figure F1: Locations of Stable equilibria for a Latin Hypercube sample of parameter space for l1=l2=1. Figure F2: Prey intake, parasite exposure, and parasite infection over the same subset of parameter space given in Figure F1. Figure F3: Locations of Stable equilibria for a Latin Hypercube sample of parameter space for l1=l2=10. Figure F4: Prey intake, parasite exposure, and parasite infection over the same subset of parameter space given in Figure F3. Figure F5: Locations of Stable equilibria for a Latin Hypercube sample of parameter space for l1=l2=100. Figure F6: Prey intake, parasite exposure, and parasite infection over the same subset of parameter space given in Figure F5. Table 1: The numbers of simulations which result in noncoexistence in each of the four scenarios (weak and strong foraging and immune trade‐offs). Figure G1: Prey intake, parasite exposure, and parasite infection over a subset of parameter space, conditional on all three species coexisting. Figure G2: The equilibrium conditions of r1a¯1 and r2a¯2 over a subset of parameter space, conditional on one species being excluded. [file EVO-75-2842-s001.zip › evo14353-sup-0011-SuppMat.png]

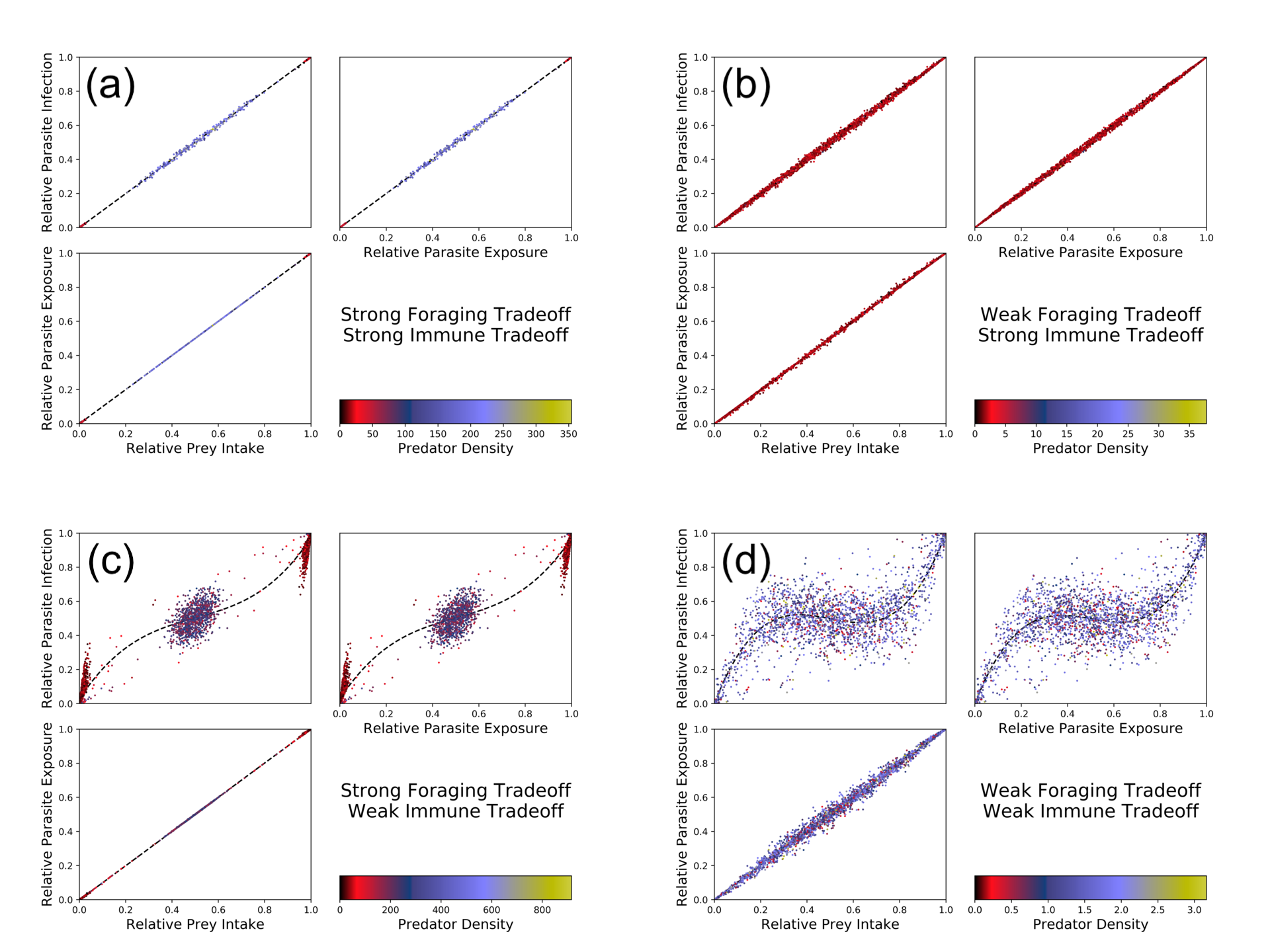

Supplement: Supplementary file 1 — Table B1: Baseline parameter values. Table B2: Figure 2 parameters. All parameters not given here are given in Table B1. Table B3: Figures 3 and 4 parameters. All parameters not given here are given in Table B1. Figure F1: Locations of Stable equilibria for a Latin Hypercube sample of parameter space for l1=l2=1. Figure F2: Prey intake, parasite exposure, and parasite infection over the same subset of parameter space given in Figure F1. Figure F3: Locations of Stable equilibria for a Latin Hypercube sample of parameter space for l1=l2=10. Figure F4: Prey intake, parasite exposure, and parasite infection over the same subset of parameter space given in Figure F3. Figure F5: Locations of Stable equilibria for a Latin Hypercube sample of parameter space for l1=l2=100. Figure F6: Prey intake, parasite exposure, and parasite infection over the same subset of parameter space given in Figure F5. Table 1: The numbers of simulations which result in noncoexistence in each of the four scenarios (weak and strong foraging and immune trade‐offs). Figure G1: Prey intake, parasite exposure, and parasite infection over a subset of parameter space, conditional on all three species coexisting. Figure G2: The equilibrium conditions of r1a¯1 and r2a¯2 over a subset of parameter space, conditional on one species being excluded. [file EVO-75-2842-s001.zip › evo14353-sup-0012-SuppMat.png]

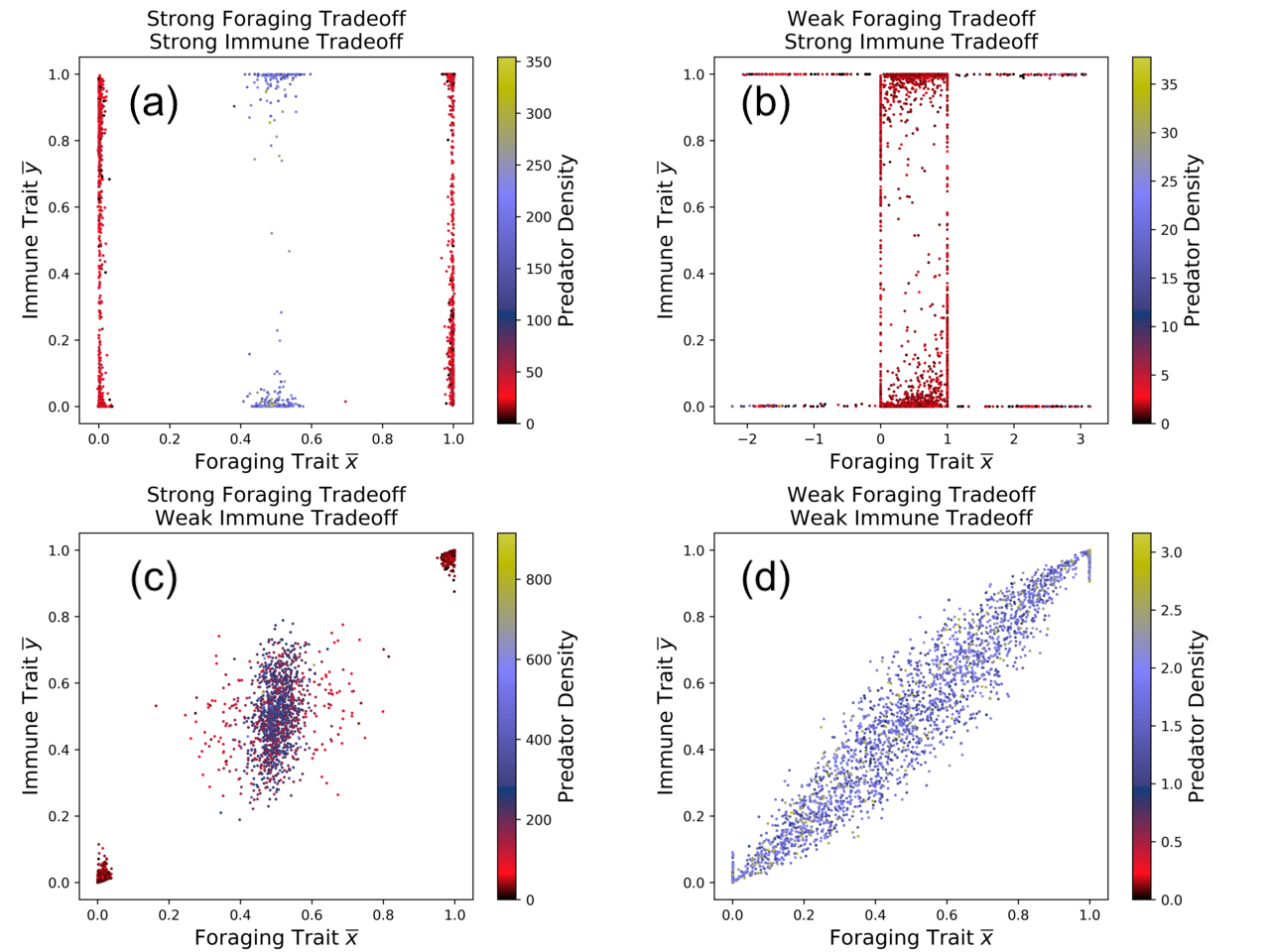

Supplement: Supplementary file 1 — Table B1: Baseline parameter values. Table B2: Figure 2 parameters. All parameters not given here are given in Table B1. Table B3: Figures 3 and 4 parameters. All parameters not given here are given in Table B1. Figure F1: Locations of Stable equilibria for a Latin Hypercube sample of parameter space for l1=l2=1. Figure F2: Prey intake, parasite exposure, and parasite infection over the same subset of parameter space given in Figure F1. Figure F3: Locations of Stable equilibria for a Latin Hypercube sample of parameter space for l1=l2=10. Figure F4: Prey intake, parasite exposure, and parasite infection over the same subset of parameter space given in Figure F3. Figure F5: Locations of Stable equilibria for a Latin Hypercube sample of parameter space for l1=l2=100. Figure F6: Prey intake, parasite exposure, and parasite infection over the same subset of parameter space given in Figure F5. Table 1: The numbers of simulations which result in noncoexistence in each of the four scenarios (weak and strong foraging and immune trade‐offs). Figure G1: Prey intake, parasite exposure, and parasite infection over a subset of parameter space, conditional on all three species coexisting. Figure G2: The equilibrium conditions of r1a¯1 and r2a¯2 over a subset of parameter space, conditional on one species being excluded. [file EVO-75-2842-s001.zip › evo14353-sup-0013-SuppMat.png]

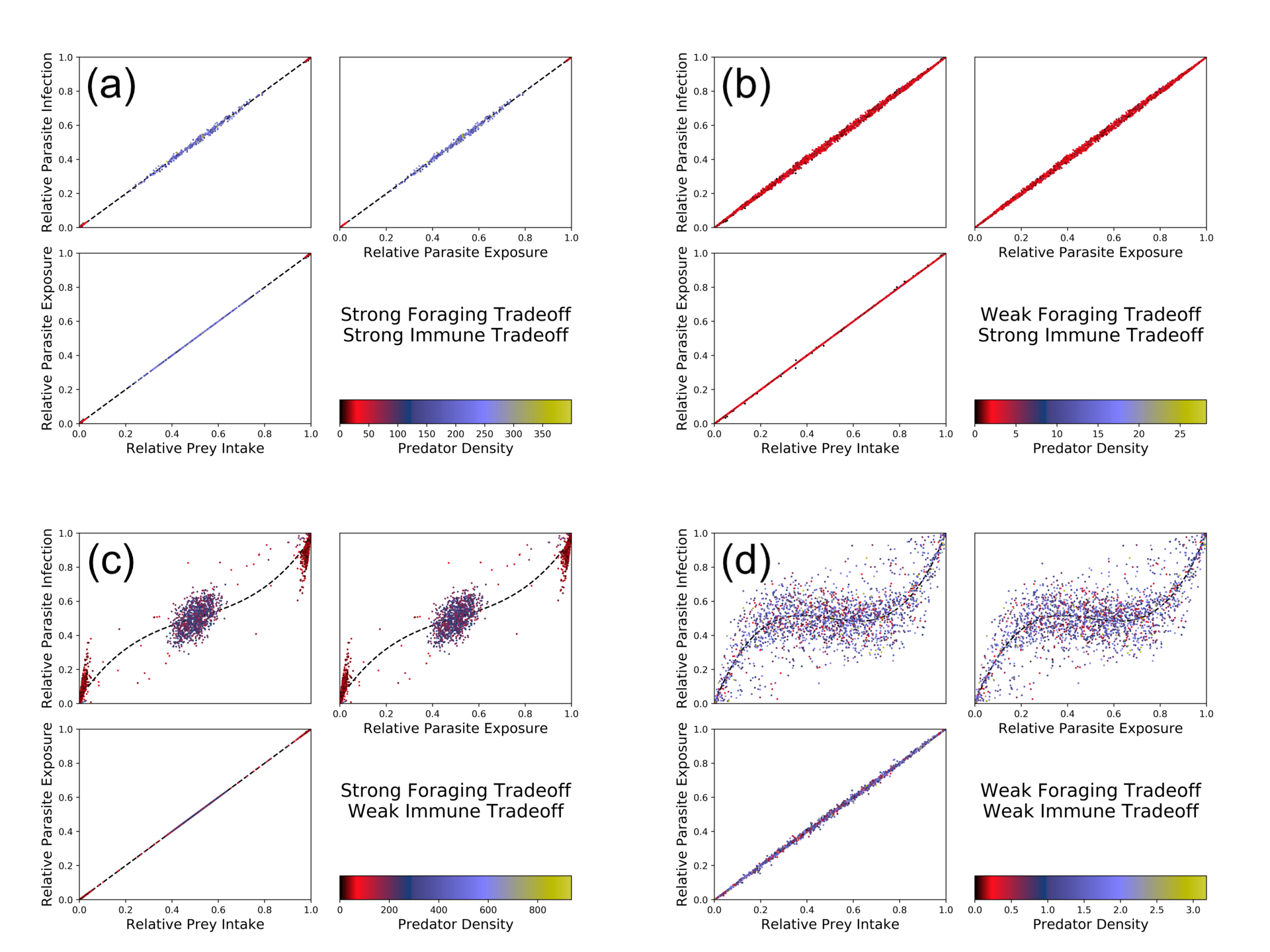

Supplement: Supplementary file 1 — Table B1: Baseline parameter values. Table B2: Figure 2 parameters. All parameters not given here are given in Table B1. Table B3: Figures 3 and 4 parameters. All parameters not given here are given in Table B1. Figure F1: Locations of Stable equilibria for a Latin Hypercube sample of parameter space for l1=l2=1. Figure F2: Prey intake, parasite exposure, and parasite infection over the same subset of parameter space given in Figure F1. Figure F3: Locations of Stable equilibria for a Latin Hypercube sample of parameter space for l1=l2=10. Figure F4: Prey intake, parasite exposure, and parasite infection over the same subset of parameter space given in Figure F3. Figure F5: Locations of Stable equilibria for a Latin Hypercube sample of parameter space for l1=l2=100. Figure F6: Prey intake, parasite exposure, and parasite infection over the same subset of parameter space given in Figure F5. Table 1: The numbers of simulations which result in noncoexistence in each of the four scenarios (weak and strong foraging and immune trade‐offs). Figure G1: Prey intake, parasite exposure, and parasite infection over a subset of parameter space, conditional on all three species coexisting. Figure G2: The equilibrium conditions of r1a¯1 and r2a¯2 over a subset of parameter space, conditional on one species being excluded. [file EVO-75-2842-s001.zip › evo14353-sup-0014-SuppMat.png]

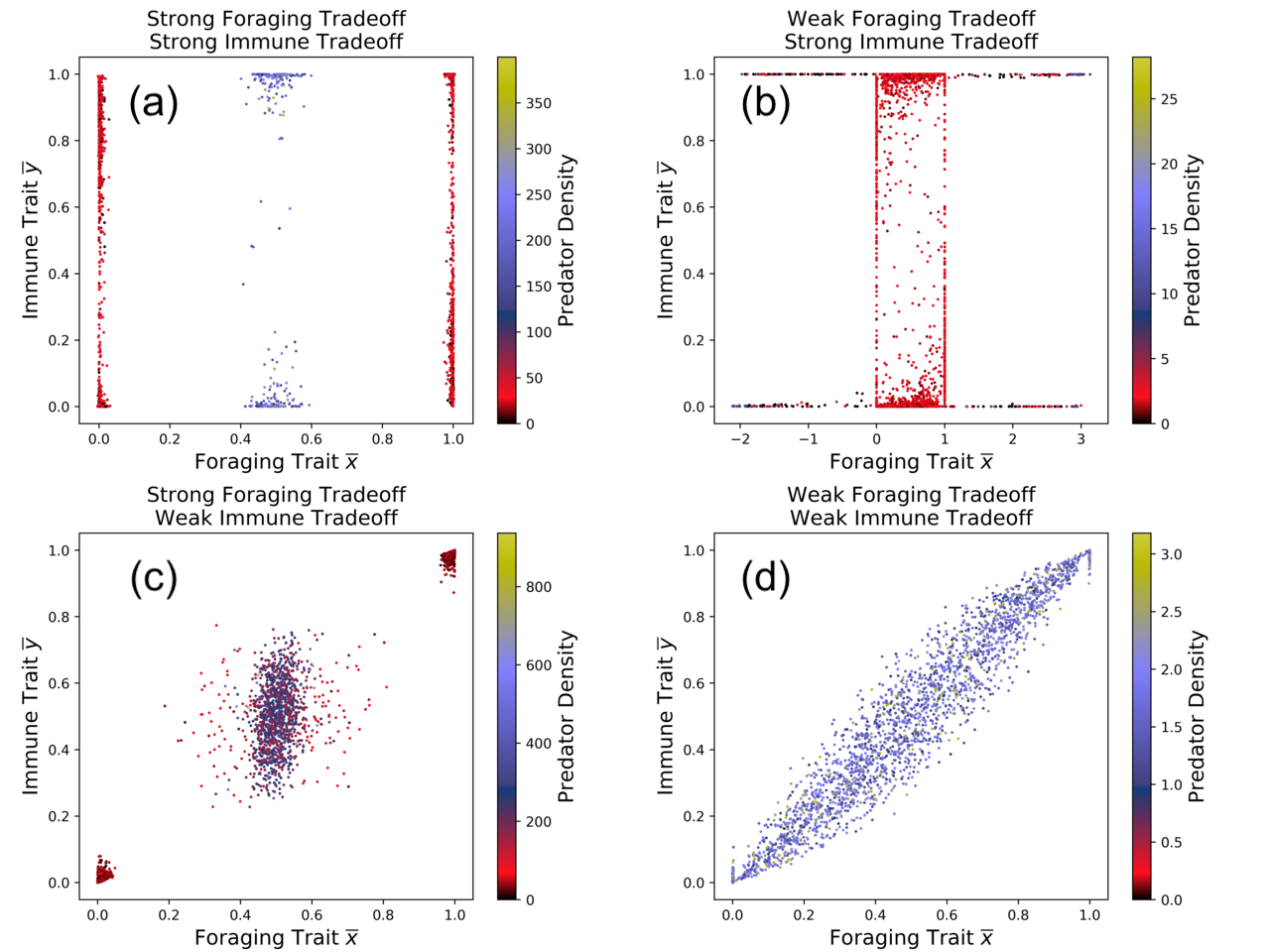

Supplement: Supplementary file 1 — Table B1: Baseline parameter values. Table B2: Figure 2 parameters. All parameters not given here are given in Table B1. Table B3: Figures 3 and 4 parameters. All parameters not given here are given in Table B1. Figure F1: Locations of Stable equilibria for a Latin Hypercube sample of parameter space for l1=l2=1. Figure F2: Prey intake, parasite exposure, and parasite infection over the same subset of parameter space given in Figure F1. Figure F3: Locations of Stable equilibria for a Latin Hypercube sample of parameter space for l1=l2=10. Figure F4: Prey intake, parasite exposure, and parasite infection over the same subset of parameter space given in Figure F3. Figure F5: Locations of Stable equilibria for a Latin Hypercube sample of parameter space for l1=l2=100. Figure F6: Prey intake, parasite exposure, and parasite infection over the same subset of parameter space given in Figure F5. Table 1: The numbers of simulations which result in noncoexistence in each of the four scenarios (weak and strong foraging and immune trade‐offs). Figure G1: Prey intake, parasite exposure, and parasite infection over a subset of parameter space, conditional on all three species coexisting. Figure G2: The equilibrium conditions of r1a¯1 and r2a¯2 over a subset of parameter space, conditional on one species being excluded. [file EVO-75-2842-s001.zip › evo14353-sup-0015-SuppMat.png]

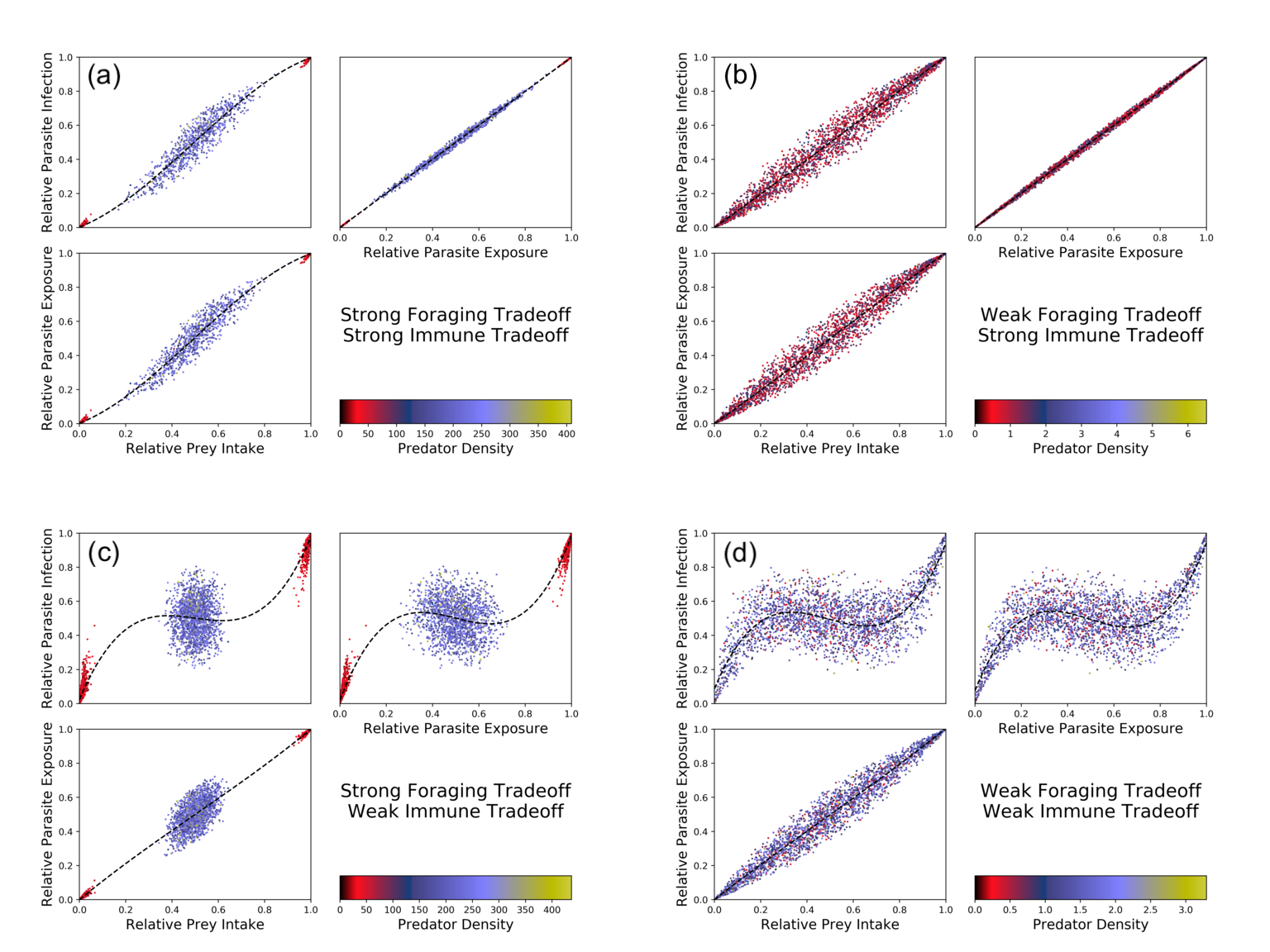

Supplement: Supplementary file 1 — Table B1: Baseline parameter values. Table B2: Figure 2 parameters. All parameters not given here are given in Table B1. Table B3: Figures 3 and 4 parameters. All parameters not given here are given in Table B1. Figure F1: Locations of Stable equilibria for a Latin Hypercube sample of parameter space for l1=l2=1. Figure F2: Prey intake, parasite exposure, and parasite infection over the same subset of parameter space given in Figure F1. Figure F3: Locations of Stable equilibria for a Latin Hypercube sample of parameter space for l1=l2=10. Figure F4: Prey intake, parasite exposure, and parasite infection over the same subset of parameter space given in Figure F3. Figure F5: Locations of Stable equilibria for a Latin Hypercube sample of parameter space for l1=l2=100. Figure F6: Prey intake, parasite exposure, and parasite infection over the same subset of parameter space given in Figure F5. Table 1: The numbers of simulations which result in noncoexistence in each of the four scenarios (weak and strong foraging and immune trade‐offs). Figure G1: Prey intake, parasite exposure, and parasite infection over a subset of parameter space, conditional on all three species coexisting. Figure G2: The equilibrium conditions of r1a¯1 and r2a¯2 over a subset of parameter space, conditional on one species being excluded. [file EVO-75-2842-s001.zip › evo14353-sup-0016-SuppMat.png]

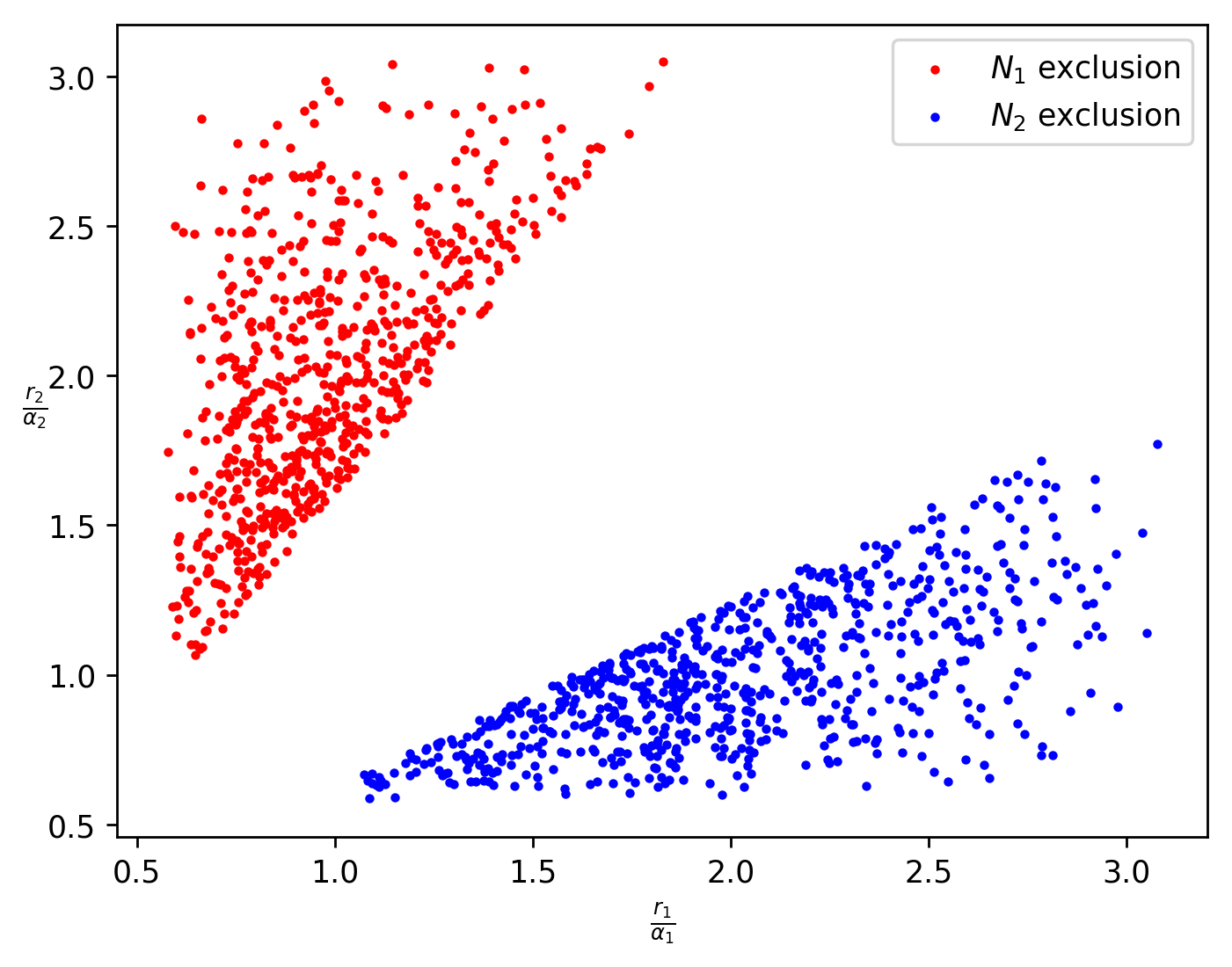

Supplement: Supplementary file 1 — Table B1: Baseline parameter values. Table B2: Figure 2 parameters. All parameters not given here are given in Table B1. Table B3: Figures 3 and 4 parameters. All parameters not given here are given in Table B1. Figure F1: Locations of Stable equilibria for a Latin Hypercube sample of parameter space for l1=l2=1. Figure F2: Prey intake, parasite exposure, and parasite infection over the same subset of parameter space given in Figure F1. Figure F3: Locations of Stable equilibria for a Latin Hypercube sample of parameter space for l1=l2=10. Figure F4: Prey intake, parasite exposure, and parasite infection over the same subset of parameter space given in Figure F3. Figure F5: Locations of Stable equilibria for a Latin Hypercube sample of parameter space for l1=l2=100. Figure F6: Prey intake, parasite exposure, and parasite infection over the same subset of parameter space given in Figure F5. Table 1: The numbers of simulations which result in noncoexistence in each of the four scenarios (weak and strong foraging and immune trade‐offs). Figure G1: Prey intake, parasite exposure, and parasite infection over a subset of parameter space, conditional on all three species coexisting. Figure G2: The equilibrium conditions of r1a¯1 and r2a¯2 over a subset of parameter space, conditional on one species being excluded. [file EVO-75-2842-s001.zip › evo14353-sup-0017-SuppMat.png]

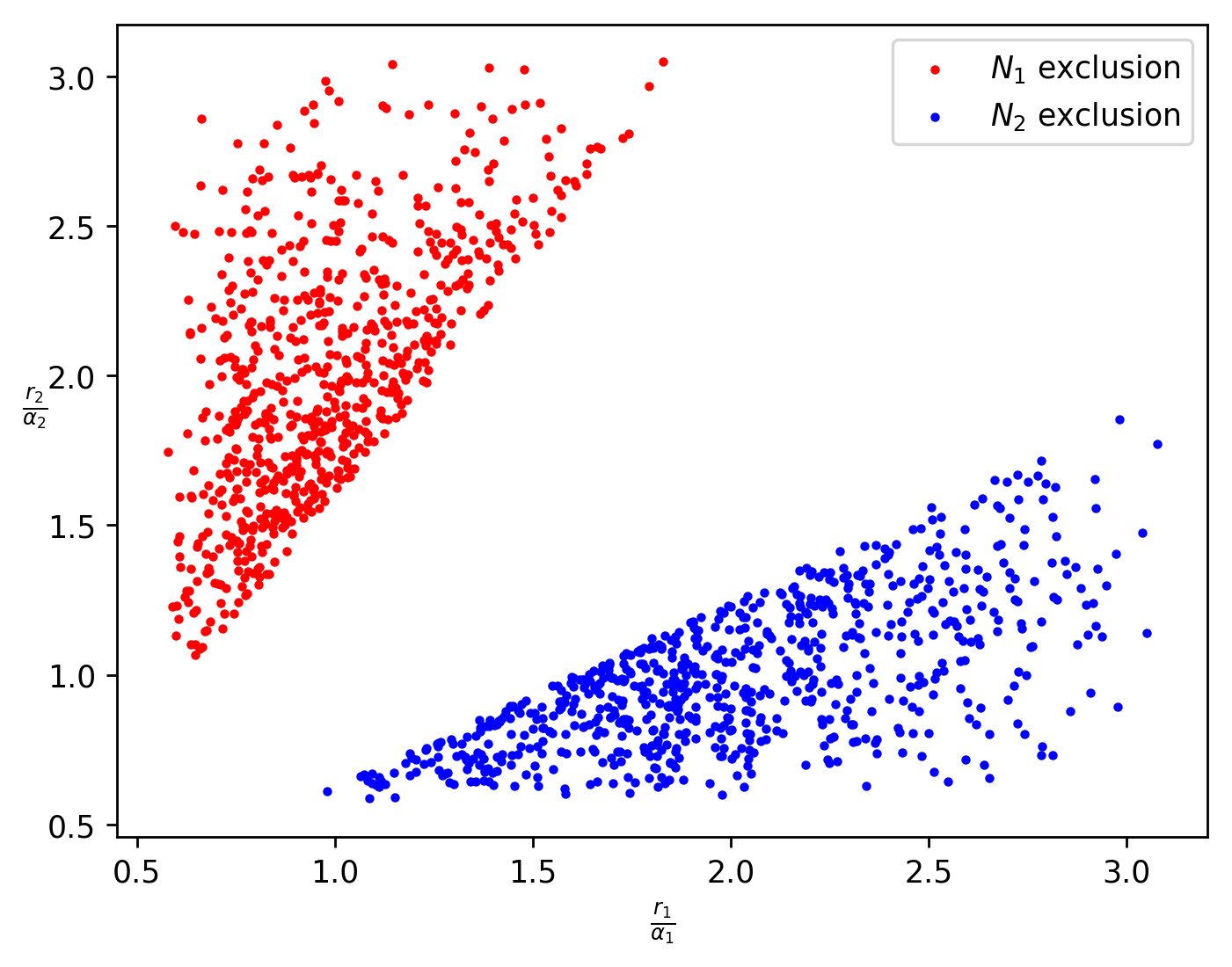

Supplement: Supplementary file 1 — Table B1: Baseline parameter values. Table B2: Figure 2 parameters. All parameters not given here are given in Table B1. Table B3: Figures 3 and 4 parameters. All parameters not given here are given in Table B1. Figure F1: Locations of Stable equilibria for a Latin Hypercube sample of parameter space for l1=l2=1. Figure F2: Prey intake, parasite exposure, and parasite infection over the same subset of parameter space given in Figure F1. Figure F3: Locations of Stable equilibria for a Latin Hypercube sample of parameter space for l1=l2=10. Figure F4: Prey intake, parasite exposure, and parasite infection over the same subset of parameter space given in Figure F3. Figure F5: Locations of Stable equilibria for a Latin Hypercube sample of parameter space for l1=l2=100. Figure F6: Prey intake, parasite exposure, and parasite infection over the same subset of parameter space given in Figure F5. Table 1: The numbers of simulations which result in noncoexistence in each of the four scenarios (weak and strong foraging and immune trade‐offs). Figure G1: Prey intake, parasite exposure, and parasite infection over a subset of parameter space, conditional on all three species coexisting. Figure G2: The equilibrium conditions of r1a¯1 and r2a¯2 over a subset of parameter space, conditional on one species being excluded. [file EVO-75-2842-s001.zip › evo14353-sup-0018-SuppMat.png]
